# A regulatory network of two galectins mediates the earliest steps of avian limb skeletal morphogenesis

**DOI:** 10.1186/1471-213X-11-6

**Published:** 2011-02-01

**Authors:** Ramray Bhat, Kenneth M Lerea, Hong Peng, Herbert Kaltner, Hans-Joachim Gabius, Stuart A Newman

**Affiliations:** 1 Department of Cell Biology and Anatomy, New York Medical College, Valhalla, NY 10595, USA; 2 Chair of Physiological Chemistry, Fakulty of Veterinary Medicine, Ludwig-Maximilians-University Munich, Veterinärstrasse13, D-80539 Munich, Germany

## Abstract

**Background:**

The skeletal elements of vertebrate embryonic limbs are prefigured by rod- and spot-like condensations of precartilage mesenchymal cells. The formation of these condensations depends on cell-matrix and cell-cell interactions, but how they are initiated and patterned is as yet unresolved.

**Results:**

Here we provide evidence that galectins, *β*-galactoside-binding lectins with *β*-sandwich folding, play fundamental roles in these processes. We show that among the five chicken galectin (CG) genes, two, CG-1A, and CG-8, are markedly elevated in expression at prospective sites of condensation *in vitro *and *in vivo*, with their protein products appearing earlier in development than any previously described marker. The two molecules enhance one another's gene expression but have opposite effects on condensation formation and cartilage development *in vivo *and *in vitro*: CG-1A, a non-covalent homodimer, promotes this process, while the tandem-repeat-type CG-8 antagonizes it. Correspondingly, knockdown of CG-1A inhibits the formation of skeletal elements while knockdown of CG-8 enhances it. The apparent paradox of mutual activation at the gene expression level coupled with antagonistic roles in skeletogenesis is resolved by analysis of the direct effect of the proteins on precartilage cells. Specifically, CG-1A causes their aggregation, whereas CG-8, which has no adhesive function of its own, blocks this effect. The developmental appearance and regulation of the unknown cell surface moieties ("ligands") to which CG-1A and CG-8 bind were indicative of specific cognate- and cross-regulatory interactions.

**Conclusion:**

Our findings indicate that CG-1A and CG-8 constitute a multiscale network that is a major mediator, earlier-acting than any previously described, of the formation and patterning of precartilage mesenchymal condensations in the developing limb. This network functions autonomously of limb bud signaling centers or other limb bud positional cues.

## Background

The formation of the vertebrate limb skeleton has become a paradigm of developmental biology due to its suitability for the study of processes such as tissue induction, cell differentiation, and spatial patterning of differentiated cell types [1]. A key cellular event in the early developing limb is the formation of condensations - high-density spot- or rod-shaped aggregates of precartilage mesenchymal cells [2-4]. These aggregates arise from and, once formed, are surrounded by, uncondensed mesenchymal cells embedded in a loose extracellular matrix (ECM). Only the condensed cells differentiate into chondrocytes. Thus condensations prefigure the limb cartilage primordia, which directly form the skeletal elements, or more usually are replaced by bone.

Although molecular and cellular changes accompanying the formation of limb precartilage condensations have been studied extensively (see [3,5], for reviews), and computational models simulating their generation *in vitro *and *in vivo *have been put forward (reviewed in [6]), the detailed mechanisms by which condensations are initiated and become arranged in spatial patterns, particularly *in vivo*, are still not fully understood. While it is well accepted that mesenchymal condensation involves local changes in cell-ECM and cell-cell adhesive interactions, some of the proposed mediators of this process, such as tenascin, NCAM and N-cadherin, have been shown not to be essential for normal limb development in mice [7-9]. Fibronectin remains a viable candidate for an indispensible ECM determinant of condensations [10-12]. But cell clusters that anticipate fully formed condensations ("protocondensations") are seen *in vitro *as early as 17 h [13], at least 12 h before fibronectin is detectable.

The proximate ECM, matricellular, and cell adhesive mediators of limb precartilage condensation must therefore be induced by molecules or cell activities that act earlier in development. While condensations are sometimes treated as downstream manifestations of a program initiated by the limb bud's signaling centers like the apical ectodermal ridge (AER) and the zone of polarizing activity (ZPA) (e.g., [14]), there is little evidence relating cellular states induced by such centers to production of the molecules that mediate cell aggregation. Moreover, formation of limb-like arrays of skeletal elements can still proceed when global positional cues are disrupted [15,16]. And whereas inducers of ECM production such as activins, TGF-*β*s and BMPs have been shown to act early in the limb chondrogenic pathway [17,18], it is unclear how they exert their effects in a patterned fashion.

In amniotes, such as birds and mammals, the formation of condensations and their differentiation occur in a proximodistal order [19]. In both the fore and hind limb buds a single proximal structure, the stylopod, develops first, followed by two parallel elements, the zeugopod, and a distal series of regularly spaced digits, the autopod. Any candidates for the early-acting determinants of precartilage condensation must be expressed in accordance with this timetable.

Here we report on the role of a class of endogenous lectins with *β*-sandwich folding known as galectins [20] in the morphogenesis and patterning of limb condensations. Attention was drawn to these molecules for their demonstrated roles in mediating cell-cell and cell-matrix interactions and eliciting potent biosignaling [21-23] as well as for the unsurpassed capacity of glycans (to which they bind) to store biological information, embodied in the concept of the sugar code (reviewed in [24]). We pursued the galectins in more detail when we found that a subset of them was expressed in a condensation-associated fashion earlier than any previously described molecules.

Galectins are frequently found within the extracellular environment (a requirement for molecular agents of tissue-scale patterning), and mediate and regulate cell-cell and cell-matrix adhesion [25], key processes in the formation of condensations. Despite the widespread occurrence of *β*-galactosides on cells, galectins are known to target distinct glycoconjugates, with their spatial organization in membrane microdomains playing a decisive role [26].

Galectins are widespread among metazoans although the number of galectins varies widely between species. In contrast to amphibians, such as *Xenopus*, which has 12 galectins [27], and mammals, where the complexity of the galectin network is even higher [28], galectin expression in the chicken is confined to five completely characterized genes [29,30]. This represents a major advantage in gaining insights on functional divergence/overlap among these homologous proteins in the limb-forming system.

The five members of the chicken galectin (CG) family are assigned to three groups with characteristic structural features: the three homodimeric proto-type proteins CG-1A, CG-1B and CG-2, most closely related to mammalian galectins-1 and -2, respectively, the chimera-type CG-3 with a collagenase-sensitive stalk, and the tandem-repeat-type CG-8 with two different lectin domains covalently connected by a linker, related to mammalian galectin-8 (for recent overviews see [29,31,32]).

CG-1A crystallizes as a homodimer with a substantial interface area [33]. In gel filtration after purification from chicken liver it runs completely as a homodimer [34], the structure of which has been confirmed by ultracentrifugation [31]. The monomeric status of CG-8 has also been confirmed by gel filtration and ultracentrifugation [29].

By comprehensive analysis we found that the genes specifying CG-1A and CG-8 are expressed at elevated levels during the initiation and early stages of condensation formation in chicken limb precartilage mesenchyme *in vitro *and *in vivo*. The corresponding proteins also appear in a condensation- and early cartilage nodule- and primordia-specific manner, with binding reactivity to the labeled CGs exhibiting the expected condensation association, but significant differences in specificity of localization.

Addition of these lectins obtained by recombinant production has dramatic and opposite effects on condensation *in vitro*, with CG-1A enhancing and CG-8 suppressing the process. These effects are borne out *in vivo*, where exogenous CG-1A induces ectopic skeletal elements and exogenous CG-8 inhibits formation of normal elements. Experiments involving inhibition of function further confirm that the two galectins are antagonists of each other. However, CG-1A and CG-8 also upregulate one another's gene expression at the mRNA level. To resolve the apparent paradox of the mutual antagonism and cooperation of these molecules we looked more closely at the direct effect of the proteins on precartilage mesenchymal cells. In a real-time turbidimetric agglutination assay we found that CG-8, though not adhesive in its own right, interferes with the direct cell-cell adhesion mediated by CG-1A. Our findings suggest that morphogenesis of individual condensations can be brought about by the action of CG-1A, but that condensation patterning depends on a multiscale local-autoactivation-lateral-inhibition network comprising CG-1A and CG-8.

## Results

### Galectin expression in limb bud mesenchyme in vitro

The previous characterization of the complete panel of CGs affords the opportunity to comprehensively study expression profiles. We first measured the relative levels of gene expression of all CGs in high density monolayer "micromass" cultures of freshly dissociated uncondensed leg precartilage mesenchymal cells by quantitative real-time PCR (qRT-PCR) of reverse-transcribed mRNA. Of the five CGs, the levels of expression of the prototype CG-1A and the tandem-repeat-type CG-8 were found to be several-fold higher than that of CG-1B and CG-2, both in the freshly dissociated cells and in the micromass cultures. The efficiencies of amplification of CG-1A, CG-1B and CG-8 were similar, and that of CG-2 about 20 percent higher (see Methods). The substantially higher amplification levels of CG-1A and CG-8 compared to the other galectins thus represent real differences in mRNA expression levels. The expression of CG-3 was negligible during the stages of condensation formation and patterning. Evidently, gene expression among CGs is differentially regulated with a preference for one of three homodimeric proteins (CG-1A) and the only tandem-repeat-type CG (CG-8). We thus focused on CG-1A and CG-8 and assayed for their expression at various points along the developmental time-table of micromass cultures, during which the mesenchymal cells undergo focal condensation and then differentiate into cartilage nodules.

**Figure 1:**
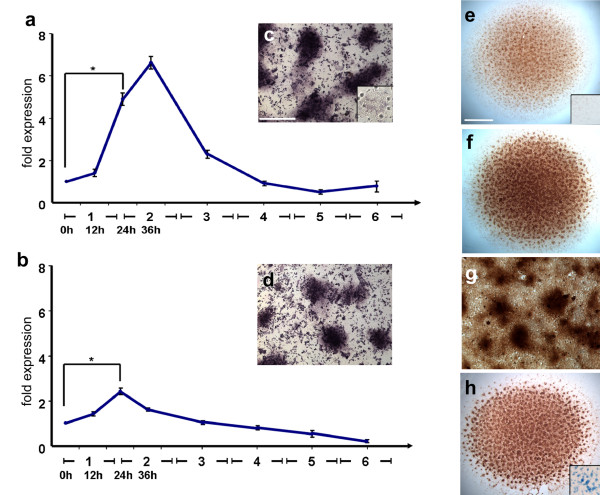
**Gene and protein expression of CG-1A and CG-8 correlates spatially and temporally with precartilage condensation pattern formation *in vitro***. (a) Relative expression of CG-1A measured by qRT-PCR at successive stages of development of leg-bud micromass cultures. (b) Relative expression of CG-8-specific mRNA measured in the same samples as (a). Differences between mRNA values at 24 h and dissociated but uncultured 5-day leg autopod precartilage cells (0 h calibrator samples) were statistically analyzed by Student's t-test. Results are shown as mean ± S.E.M. (* p < 0.05). (c, d) Gene expression of (c) CG-1A and (d) CG-8 in 2-day leg micromass cultures, assayed by in situ hybridization, is spatially coincident with condensations. Images are representative of two independent results. Inset in (c), condensation at same magnification from no-probe control culture. (e-h) Spatial localization of CG-1A protein *in vitro *by indirect immunostaining at (e) 15 h, (f, g) day 3 (low and high magnification, respectively) and day 6 (h). Inset in (e), region at same magnification from center of control culture in which no primary antibody was applied. Inset in (h), region at same magnification from 6 day culture stained for cartilage with Alcian blue. Images (c), (d) and (g) are at the same magnification; scale bar in (c) represents 0.2 mm. Images (e), (f) and (h) are at same magnification; scale bar in (e) represents 1 mm.

CG-1A gene expression increased about 7-fold within the first two days of culture, i.e., during the period of condensation formation (Figure 1a). The CG-8 mRNA level rose as well, but not as sharply as that of CG-1A (~2.5 fold) (Figure 1b). Message levels of both then steadily declined to about 15% (CG-1A) and 20% (CG-8) of their expression maxima by the fifth and sixth day of cultures, by which time the condensations had differentiated into nodules of cartilage. The period of condensation patterning and morphogenesis was thus temporally associated with the maximal expression levels of both CG-1A and CG-8.

We next visualized the spatial distribution of mRNAs specific for CG-1A and CG-8 *in vitro *by *in situ *hybridization of 2-day cultures with gene-specific digoxigenin-labeled riboprobes. CG-1A and CG-8 mRNAs were both detected with strong signal intensity within cells that were part of the condensations, as well as in a small number of scattered, isolated cells in the intercondensation mesenchyme (Figure 1c,d). Control cultures, incubated without a probe or with "sense" riboprobes, failed to show any staining, confirming the specificity of the hybridization reaction.

The presence of CG-1A protein in leg bud micromass cultures was monitored at various stages of development (i.e., uncondensed, condensing, condensed and differentiating, differentiated, etc.) by indirect immunostaining using an antibody preparation free from cross-reactivity to any other CG. CG-1A was strongly localized in a spot-like pattern in cultures in which the condensations were in the process of forming (day 1; Figure 1e), already formed (day 3; Figure 1f,g), or had already differentiated into cartilage (day 6; Figure 1h). The immunostaining for CG-8 protein *in vitro *had a similar nodular pattern (Additional file 1, Figure S1a,b). The intensity of the staining increased and its spatial localization became more specific as the condensations grew larger and the cultures matured.

### Galectins are early and specific markers of precartilage condensations

**Figure 2:**
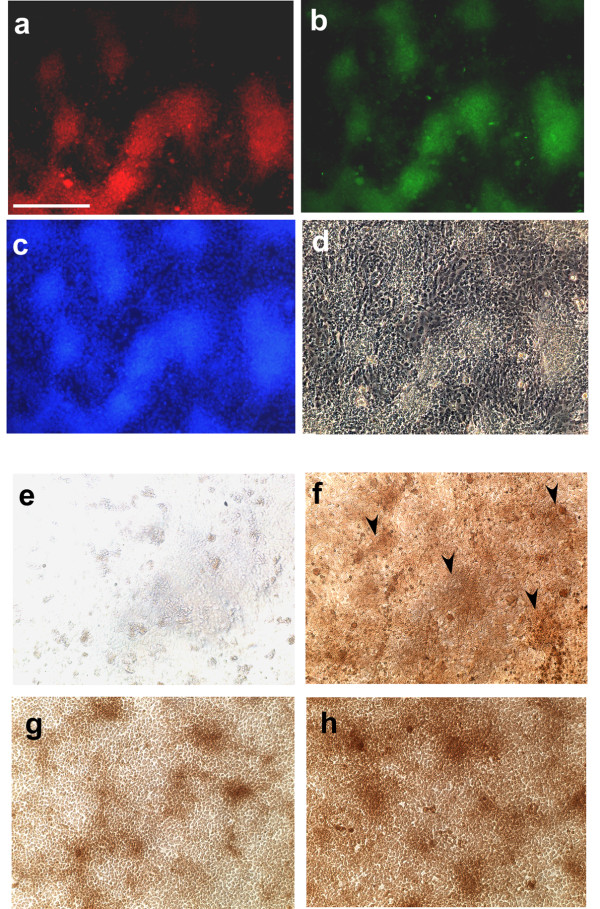
**CG-1A is an early endogenous marker for limb precartilage condensations *in vitro***. (a-d) A microscopic field in a 2-day leg micromass culture showing (a) indirect immunolocalization of CG-1A, (b) staining with FITC-conjugated peanut agglutinin (PNA); and (c) DAPI. The same field was photographed under (d) phase-contrast microscopy. (e-f) 9-hour fixed cultures incubated with (e) HRP-conjugated PNA fail to show staining but when immunostained for (f) CG-1A show patchy "proto-condensations" at 9 h of incubation (black arrowheads). (g-h) 18-hour fixed cultures incubated with (g) HRP-conjugated PNA or immunostained for (h) CG-1A show fully formed condensations of similar spatial patterns. Images (a-h) are at same level of magnification; scale bar in (a) represents 0.2 mm.

In order to confirm that the galectins were specifically localized to the condensations, micromass cultures that had been incubated for two days and subsequently fixed for staining were treated with anti-CG-1A antibody and with Dylight 594-conjugated goat anti-rabbit secondary antibody to monitor localization of CG-1A (Figure 2a). The same cultures were processed with FITC-labeled peanut agglutinin (PNA; Figure 2b), a plant lectin, and with DAPI to mark the nuclei (Figure 2c). The glycan moiety to which PNA binds is a known marker for precartilage mesenchymal condensations [35]. We found that CG-1A and glycans reactive with PNA colocalized in the 2-day condensations. The same field viewed by phase-contrast optics (Figure 2d) showed that the stained condensations were surrounded by non-condensed mesenchyme, which, significantly, stained very sparsely with PNA and for CG-1A.

Monitoring the reactivity with PNA is one of the earliest-described methods for detection of limb precartilage mesenchymal condensations *in vivo *and *in vitro *[35,36]. Both PNA and the antibody against CG-1A stained the earliest detectable condensations at 18 h of incubation (Figures 2g,h). Staining for CG-1A as well as CG-8 was also observed as early as 9 h of incubation (Figures 2f, Additional file 1, Figure S1c). Although morphological condensations have not yet appeared in such early cultures, the CG-1A- and -8-dependent staining is enhanced compared to the surrounding mesenchyme in patches of a similar spatial scale to condensations. We infer that these patches, which at this stage are only relatively, not specifically, enriched in CG-1A and CG-8, are the early primordia of the future condensations, and ultimately cartilage nodules. These "proto-condensations" are not reactive with PNA (Figure 2e at 9h), making CG-1A and CG-8 the earliest markers thus far reported for condensing precartilage mesenchyme.

In order to determine if the enhanced staining of CG-1A/-8 in condensations was due to increased protein level in condensed cells or an artifact of higher density at these sites, we co-immunostained cultures for each of the two CGs and for cell nuclei, using DAPI. The ratio between mean fluorescence intensity within and outside condensations was calculated individually for the galectins and for DAPI. The mean intensity of CG-1A and CG-8 fluorescence in condensation mesenchyme after normalization to intensity of their staining in non-condensed cells was 3.5- and 2.5-fold that of DAPI fluorescence in condensations, respectively after the latter had been normalized to staining of non-condensed cell nuclei (Additional file 1, Figure S1d,e). Thus, both galectins were preferentially present in cells that participated in leg condensation morphogenesis. Similar results were obtained with myoblast-free wing bud mesenchyme (not shown).

### CG-1A and CG-8 are expressed in condensations of developing limb buds

**Figure 3:**
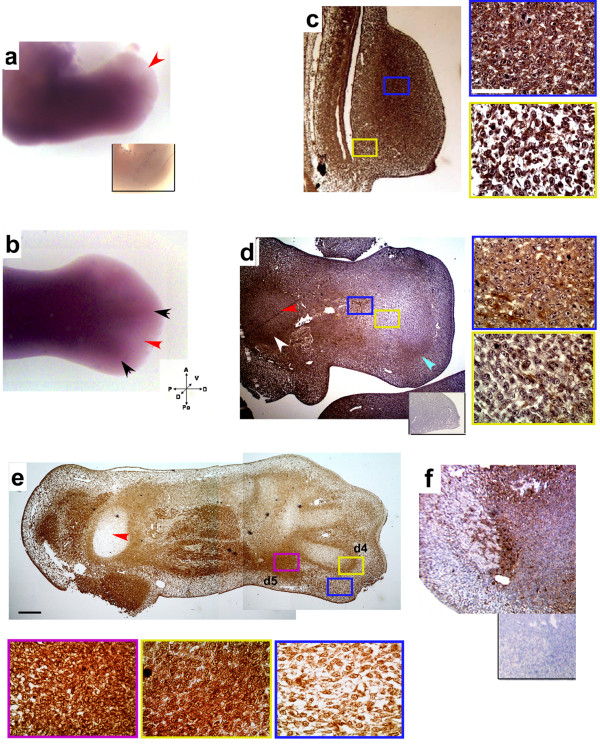
**CG-1A is localized to developing chicken leg bud precartilage condensations**. (a) Staining by in situ hybridization for CG-1A mRNA in the core of a 5-day leg bud with the distal-most cells underlying the AER unstained (red arrowhead). Inset shows control 5-day leg bud with probe omitted. In a 6-day leg (b), digits stain for CG-1A mRNA (black arrowheads) but interdigits are unstained (red arrowhead) (c) Indirect immunostaining for CG-1A protein in the 3½-day leg bud stylopod is predominantly extracellular (high-magnification view of the field enclosed by the blue box). Flank cells are sparsely stained (yellow box). (Scale bar: 50 *μ*m). (d) In a 5-day leg section there is staining for CG-1A in the stylopod perichondrium (white arrowhead) but not its core (red arrowhead). In the zeugopod, the two intensely stained primordia (blue box) are separated by a region of unstained cells (yellow box). The developing autopodium is unstained except for a crescent representing the fourth digit condensation (blue arrowhead). Inset shows leg section with primary antibody omitted. (e) The peristylopod mesenchyme in a 6-day leg section stains for CG-1A, but not the core (red arrowhead). Fusiform cells in the intensely stained mid-leg were confirmed in companion sections to be myoblasts. Strong CG-1A staining is seen in the perichondria and leading edges of the third and fourth digits (yellow box), and in the ultimately regressing "fifth" digit condensation (violet box). Interdigit cells as well as those subjacent to the ectoderm stain sparsely (blue box). The fourth and fifth digits are marked as d4 and d5. (f) 5-day wing stylopod, showing intense CG-1A staining in the humeral crescent. Inset shows a comparable region (at lower magnification) of a control wing with primary antibody omitted. (Scale bar (e): 1 mm). Pull-out images were photographed using a 63× objective (Scale bar: 50 *μ*m).

Using labeled CG-1A- and CG-8-specific riboprobes, the spatial organization of mRNA production was assayed in leg buds of 5-6-day chicken embryos. In 5-day leg buds mRNAs of both CG-1A and CG-8 were concentrated strongly in the zeugopod region, staining both the premuscle masses and cartilage primordia. In contrast, staining was relatively sparse subjacent to the apical ectodermal ridge (AER) (Figure 3a, Additional file 2, Figure S2a). In 6-day leg buds the staining for digit primordia made them discernible from the relatively clear interdigital tissue (Figures 3b, Additional file 2, Figure S2b). This is consistent with our *in vitro *findings (Figure 1c), where the condensations contained CG-1A-specific mRNA, while the pericondensation mesenchyme did not.

In sections of early developing leg buds at 3½-days (Figure 3c), CG-1A protein was present throughout the mesoblast (Figure 3c, blue box), but sparse in the somatopleural mesenchyme underlying the leg bud and the contiguous flank (Figure 3c, yellow box), and in the region subjacent to the AER. The spatial pattern of CG-8-specific staining in 3½-day leg sections was similar to that of CG-1A (Additional file 2, Figure S2c).

In 5-day leg-bud sections (Figure 3d), CG-1A-specific staining was more intense in the tibiotarsal (blue box) and fibular condensations than in the surrounding uncondensed mesenchyme (yellow box). The prospective autopod area was relatively devoid of stain except for a crescent-shaped focus in the posterior part, representing the condensation of the fourth digit (blue arrowhead). The corresponding regions of 5-day limbs stained less distinctly for CG-8 (Additional file 2, Figure S2d). The perichondrium and the outer circumferential part of the stylopod also showed positivity for both CG-1A (Figure 3d, white arrowhead) and CG-8 (Additional file 2, Figure S2d, white arrowhead).

In 6-day leg bud sections stained for CG-1A protein (Figure 3e), the stylopod, which had been sectioned along a plane orthogonal to its long axis, showed very sparse staining in its central region (Figure 3e, red arrowhead), which consisted of cartilage at this stage. Premuscle masses in the mid-leg region showed intense staining of fusiform cells for CG-1A. In the autopod region, the third and fourth digits could clearly be discerned on the basis of intense CG-1A staining (Figure 3e, yellow box) and moderate staining for CG-8 in their distal crescents (Additional file 2, Figure S2e, yellow box, crescent-shaped signaling centers in the most distal part of the digits; [18]), in the nascent joints at their proximal ends, and in the perichondria (Additional file 2, Figure S2e).

Perichondrial and digital crescent staining of both CG-1A and CG-8 rendered the digit primordia discernible from the non-condensing tissue between the leg bud ectoderm and the precartilage primordia, and interdigital mesenchyme, which was not positive for either CG-1A or CG-8 (Figure 3e, blue box and Additional file 2, Figure S2e, blue box, respectively). Staining was strong in the prospective region of the "fifth digit" (Figure 3e, violet box, for CG-1A). Here, the precartilage cells condense and differentiate into cartilage only to regress later [37]. Digital primordia (yellow and violet boxes) show round mesenchymal cells and no fusiform myoblasts or tendinocytes. The *in vivo *staining of CG-1A is consistent with our *in vitro *results. That is, the interdigit and interstitial mesenchyme, which corresponds to the intercondensation mesenchyme *in vitro*, stained minimally compared to the intense CG-1A signal and moderate CG-8 staining intensity in prospective and differentiated cartilage primordia *in vivo *and in condensations and nodules *in vitro*. The "humeral crescent" and some of the differentiated chondrocytes in the stylopod region of a 5-day wing bud stained heavily for CG-1A (Figure 3f), also consistent with *in vitro *results on myoblast-free wing mesenchyme.

### Binding reactivity for CG-1A and -8 is generally localized to condensations

**Figure 4:**
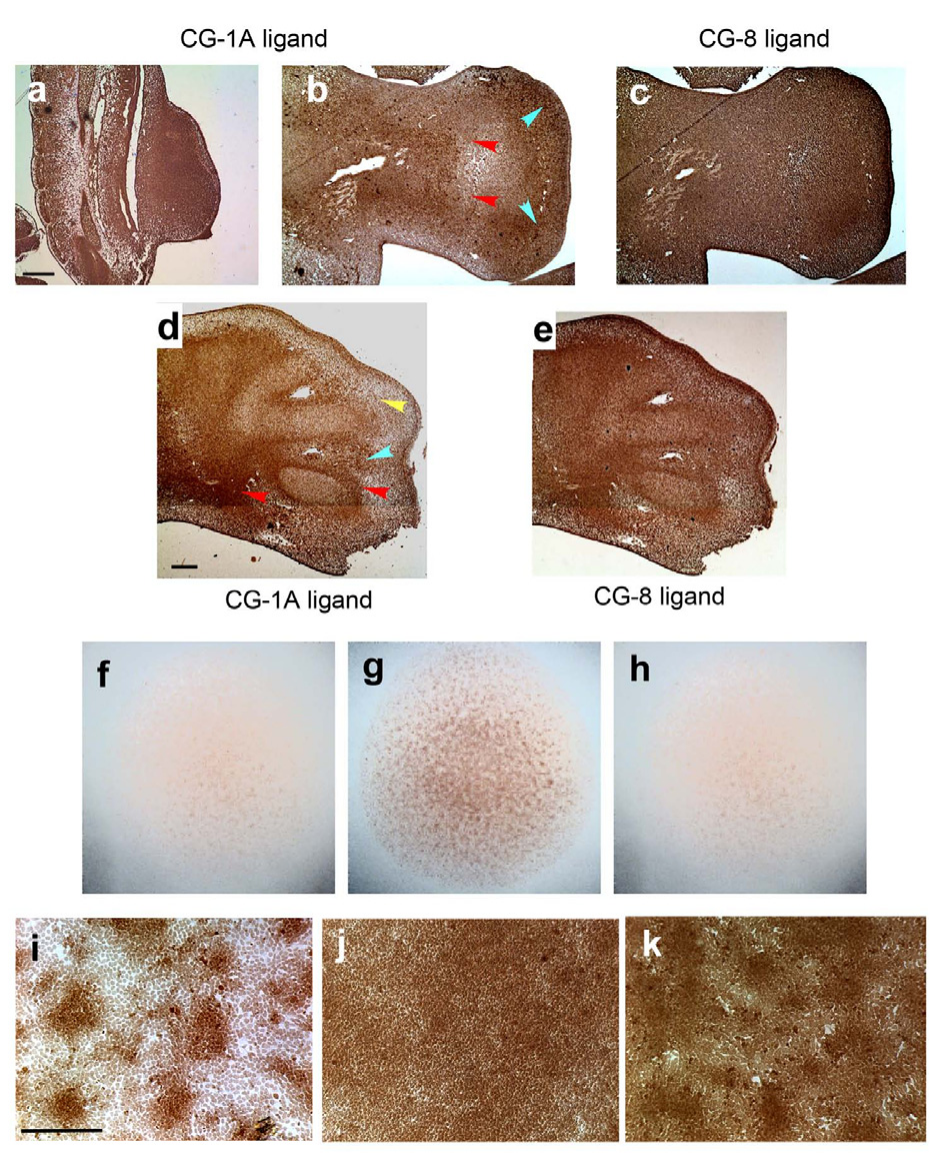
**Dynamics of the CG-1A and -8 binding reactivity in chicken leg bud mesenchyme**. (a) Staining by biotinylated CG-1A for its binding sites in a 3½-day chicken leg section. (b) Staining for CG-1A binding reactivity in 5-day leg sections in the zeugopod (red arrowheads) and in the autopod (blue arrowhead) and a more diffuse spatial localization of CG-8 binding reactivity (c). (d) Staining showing CG-1A binding reactivity in a 6-day leg-bud in the prospective digit primordia (red arrowheads), moderate staining in the interdigital mesenchyme (blue arrowhead) and lack of staining in the interstitial mesenchyme (yellow arrowhead), and (e) more diffuse staining for CG-8 binding reactivity. Images (a-c) have the same scale, as do images (d) and (e), with the scale bars in (a) and (d) representing 1 mm. (f-h) Micromass cultures of leg bud mesenchyme at 15 h of incubation. The culture in (g) shows the spatial pattern of CG-1A binding reactivity using a biotinylated CG-1A probe. The two flanking panels show control cultures. In (f) the biotinylated CG-1A probe was omitted and in (h) the culture was treated with probe that had been pre-incubated overnight with 50 mM lactose. Diameter of the micromass cultures, ~3 mm. (i) Condensation-specific expression of CG-1A-reactive sites in a 2-day leg micromass culture. (j) Addition of 10 *μ*g/ml CG-1A increases extent of its reactivity and expands its localization to precondensation cells, in contrast to the nodular pattern seen in the untreated culture (i). (k) Staining profile of CG-8-reactive sites with biotinylated CG-8 in 2-day leg micromass culture show them to be present both outside and inside condensations. The staining within the condensations is more intense than in the pericondensation regions. Images (i-k) are at the same magnification; scale bar in (i) represents 0.2 mm.

Using biotinylated CG-1A and CG-8 as probes, the spatial localization of accessible sites for CG-1A and CG-8 were probed in leg-bud sections at various developmental stages. In early 3½-day leg sections, the staining of CG-1A-binding sites closely corresponded spatially with the localization of the proteins; in particular, they were found throughout the core of the leg bud and were absent in the flank region and subjacent to the AER (Figure 4a). In 5- and 6-day leg sections the spatial localization of CG-1A binding reactivity corresponded generally with that of CG-1A itself (Figure 4b,d) with intense staining in cartilage primordia, digit perichondria and *in vivo *condensations (red arrowheads) relative to interstitial and subridge mesenchymal cells (yellow arrowhead). However, the interdigit cells also stained almost as intensely as the digit perichondria (blue arrowhead). The staining for accessible sites of CG-8 binding (Figure 4c, 5-day leg section; and 4e, 6-day leg section) was more uniform.

*In vitro*, binding of biotinylated CG-1A was in evidence by about 15 hours of culture (Figure 4g) and localized strongly to condensations in 2-day cultures (Figure 4i). This raised two questions: (i) which is the first to exhibit patterned expression, CG-1A or the ligand(s) to which it binds? and (ii) does one of these molecules influence the expression of the other? Whereas CG-1A stains protocondensations in 9-hour cultures (Figure 2f), the staining for accessible binding sites at this stage was very sparse (data not shown). Treatment of cultures with exogenous CG-1A, however, increased the area of reactivity and the staining intensity (Figure 4j).

The staining with biotinylated CG-8 as probe was more diffuse than that with labeled CG-1A, with a discernible presence in non-condensing cells and slightly more intense staining in condensations (Figure 4k). In contrast to staining for CG-8 protein (see above) the higher density of cells in the condensations probably accounts for the slightly higher staining intensity of CG-8 binding activity in these foci. Treatment of cultures with exogenous CG-8 had no effect on the spatial pattern of accessible CG-8-binding sites (not shown). To determine whether the detected binding was dependent on the lectin site we tested the effect in presence of lactose. Indeed, the binding reactivity was inhibited when the fixed cultures were incubated with a mixture of biotinylated galectins and lactose (Figure 4h).

### CG-1A increases condensation number and causes fusion of condensations

**Figure 5:**
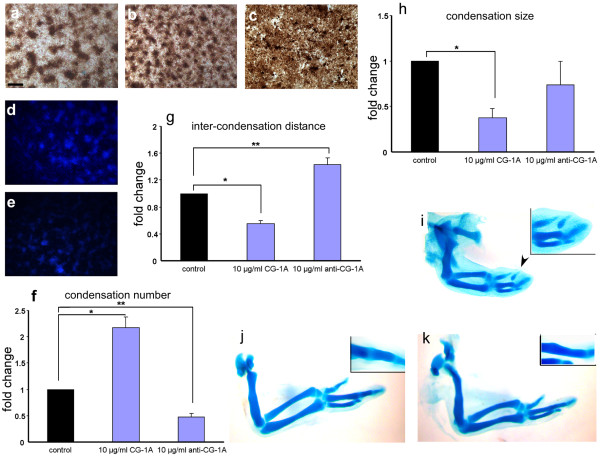
**Addition of CG-1A increases condensation number and density *in vitro *and induces ectopic cartilage *in vivo***. (a) PNA staining of a 2-day control leg culture. (b) Addition of 10 *μ*g/ml CG-1A increases the condensation number, with a concomitant decrease in size. (c) Treatment with 20 *μ*g/ml CG-1A causes the condensations to fuse. (d) Untreated control 2-day micromass culture fixed and stained with DAPI. (e) Culture treated with 10 *μ*g/ml anti-CG-1A antibody, fixed at day 2, and stained with DAPI. Photomicrographs (a-e) are at same level of magnification; bar in (a) represents 0.25 mm. (f) Graph showing change in condensation number upon addition of 10 *μ*g/ml CG-1A and 10 *μ*g/ml anti-CG-1A antibody, respectively. (g) Graph showing change in inter-condensation distance upon addition of 10 *μ*g/ml CG-1A and 10 *μ*g/ml anti-CG-1A antibody, respectively. (h) Graph showing change in condensation size upon addition of 10 *μ*g/ml CG-1A and 10 *μ*g/ml anti-CG-1A antibody, respectively. All three variables (condensation number, size and inter-condensation distance) were statistically analyzed using one-way analysis of variance comparing means by the Tukey-Kramer post-hoc analysis. Results are shown as means ± S.E.M. (* p < 0.05). (i) 5½ day wing bud injected on with CG-1A, fixed and stained with Alcian blue on day 8, shows the presence of an ectopic digit in the site of injection (black arrowhead). Inset of i shows the ectopic digit in detail. (j) Untreated 8½ day control wing stained with Alcian blue showing three digits; with discernible patency of joint space between the second digit phalange and 1^st ^metacarpal in the inset of (j). (k) Contralateral wing bud of (j), injected with CG-1A on the 5^th ^day, fixed and stained with Alcian blue showing fusion of the phalange and 1^st ^metacarpal of the second digit and obliteration of their joint space (inset of k).

Probing the tissue level and cellular localization of CG-1A shows that it is mostly present on the surfaces of and between cells within the precartilage mesenchymal condensations *in vitro *and *in ovo *[38]. This pattern suggests that it may have a role in the morphogenesis of the condensations. To address this, we added CG-1A in increasing amounts to freshly prepared leg micromass cultures and assayed for alteration in condensation patterning by staining the cultures fixed on the third day with PNA. Below 5 *μ*g/ml CG-1A, there was no discernible change in pattern. At 10 *μ*g/ml, the number of condensations increased and their mean size decreased (Figure 5b) compared to untreated control cultures (Figure 5a). When the concentration was increased to 20 *μ*g/ml, adjacent condensations began to fuse (Figure 5c). Addition of CG-1A did not increase the diameter of the micromass cultures, nor did it lead to overgrowth of the monolayered cells.

We also took advantage of the blocking capacity of the antibody preparation. Cultures treated with 10 *μ*g/ml polyclonal anti-CG-1A antibody, grown for three days, fixed and stained with DAPI to visualize the condensation pattern, exhibited a decrease in both the size and number of condensations, and the condensations were spaced further apart (Figure 5e, compare with untreated control culture Figure 5d).

Using a separate approach, small interfering (si)RNA oligonucleotides directed against CG-1A-specific mRNA were transfected by lipofection into cells of freshly prepared leg mesenchymal cultures. These cultures were grown for three days along with mock-treated counterparts, using oligonucleotides with a scrambled sequence, and these were also fixed and stained with DAPI. The decrease in expression level of CG-1A due to silencing was assessed both by indirect immunofluorescence (Additional file 3, Figure S3a,b and c show CG-1A staining control, of siRNA-treated and of mock-transfected micromass cultures, respectively) and by qRT-PCR (Additional file 3, Figure S3d, target mRNA levels averaged 1/4 of control values). There was no change in condensation pattern between the control using mock transfection and untreated cultures. In contrast, condensations in siRNA-treated cultures decreased in number as well as in size, and were spaced further apart (Additional file 3, Figure S3e,f show DAPI stained untreated and siRNA-treated cultures, respectively).

In order to quantitate the effect of CG-1A addition and inhibition (by siRNA or blocking antibody) *in vitro*, three variables were measured: mean condensation number, mean condensation size and mean inter-condensation distance. The condensation number upon CG-1A addition and inhibition was 2.2-fold and 0.5-fold that of control, respectively (Figure 5f). The mean inter-condensation distance upon CG-1A addition was 0.5-fold that of control and upon anti-CG-1A antibody addition, 1.5-fold that of control (Figure 5g). The mean condensation size with CG-1A addition was lower (0.4-fold control size), whereas with CG-1A inhibition (by antibody treatment) it was not significantly altered (Figure 5h). The change in condensation number (Additional file 3, Figure S3g) and size (Additional file 3, Figure S3h) after CG-1A knockdown by RNAi were consistent with CG-1A antibody treatment, although, unlike the latter, the former did not significantly alter the mean inter-condensation distance (Additional file 3, Figure S3i).

To determine whether our *in vitro *results had relevance *in vivo*, CG-1A was injected between days 5 and 6 into the wings of 10 chicken embryos. The injections were performed between the second and third digit primordia (the interdigit mesenchyme at this stage still retaining digit-forming potential; [39]) and also in the prospective digit mesenchyme. The right wings of 10 control 6-day embryos were subjected to injections of bovine serum albumin (BSA). By 8½ days, the cartilage pattern visualized by Alcian blue staining showed no change in either the control untreated (Figure 5j) or BSA treated wings. Of the 10 experimental embryos, seven survived, and there was no change in wing phenotype in one of them. In four of the CG-1A-injected wings, however, there was an ectopic cartilage element between the second and third digit primordia (Figure 5i; fixed at 8 days). In the other two wings fusion between the phalange and the metacarpal was observed (Figure 5k; inset shows obliteration of the joint space between the two cartilage elements). There were no such fusions in control 8½-day wings (Figure 5j; inset showing patent joint space between metacarpal and phalange).

CG-1A siRNA (or its scrambled control oligo) mixed with the transfection agent Lipofectamine was injected into the uncondensed wing autopod field of 5-day embryos, which were grown until 7½ days, fixed and stained for cartilage. The wings of five embryos injected with control oligo were all similar in cartilage morphogenesis to the untreated counterparts. Injection of the CG-1A siRNA into the autopod, however, resulted in lack of any digital primordia (Additional file 3, Figure S3j) in comparison to the developing cartilage primordia in the counterpart untreated wings (Additional file 3, Figure S3k) in three of four embryos in which the injections were made.

### Treatment with exogenous CG-8 decreases condensation number and size

**Figure 6:**
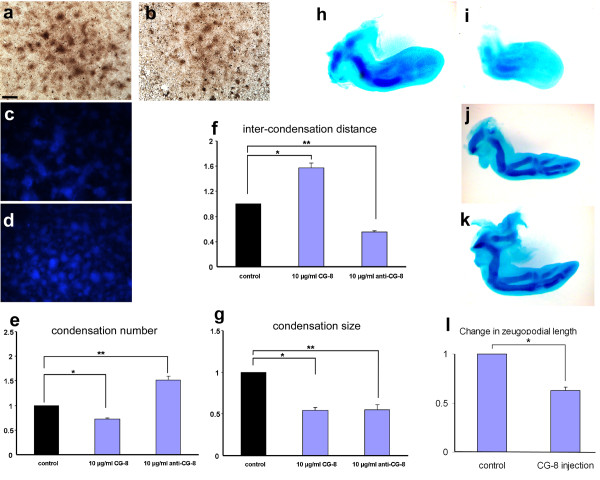
**Addition of CG-8 decreases condensation number and density *in vitro *and impairs digit development *in vivo***. (a) Staining by PNA of a 2-day control leg culture (b) Culture treated with 10 *μ*g/ml CG-8, showing a decrease in number of condensations, with a concomitant and uniform decrease in their sizes. (c) Control 2-day culture stained with DAPI. (d) Culture treated with 10 *μ*g/ml rabbit polyclonal anti-CG-8 antibody, fixed at day 2, and stained with DAPI. (e) Graph showing change in condensation number upon addition of 10 *μ*g/ml CG-8 and 10 *μ*g/ml polyclonal anti-CG-8 antibody, respectively. Photomicrographs (a-e) are at same level of magnification; scale bar in (a) represents 0.25 mm. (f) Graph showing change in inter-condensation distance upon addition of 10 *μ*g/ml CG-8 and 10 *μ*g/ml polyclonal anti-CG-8 antibody, respectively. (g) Graph showing change in condensation size both upon addition of CG-8 and polyclonal anti-CG-8 antibody, respectively. The three variables (mean condensation number, mean size and mean inter-condensation distance) were compared as in the legend to Figure 5. (h-i) Wing bud (i) injected with CG-8 on 5^th ^day of incubation, fixed and stained with Alcian blue, reveals absence of any cartilage primordia in the autopod and poorly stained, smaller zeugopodial cartilage in comparison with (h) control left wing. (j-k) Right wing bud injected on 5^th ^day of incubation with (j) CG-8 and (k) contralateral untreated control wing bud, both stained with Alcian blue at day 9, showing decrease in length of zeugopodial primordia due to CG-8 treatment. (l) Graph showing decrease in zeugopodial cartilage primordium length when CG-8 was injected into 5-day wing buds. The primordium lengths were analyzed using one-way analysis of variance comparing means by the Tukey-Kramer post-hoc analysis. Results are shown as mean ± S.E.M. (* p < 0.05).

Leg bud micromass cultures were treated with 10 *μ*g/ml CG-8, grown for 3 days, and fixed and stained with PNA. In these cultures, there was a decrease in the number and size of the condensations (Figure 6b) compared to control cultures (Figure 6a). The mean condensation number of treated cultures was 0.75-fold that of the control value (Figure 6e), and the mean size of the condensations and the mean inter-condensation distance were 0.5-fold (Figure 6f) and 1.6-fold (Figure 6g) that of controls, respectively. Although we found that addition of exogenous CG-8 at several-fold the concentration used here induces apoptosis in these cultures, we saw no evidence of reduction in the diameter of the micromasses nor denuded areas in the micromass cell monolayer with this treatment.

Treatment of cultures with 10 *μ*g/ml anti-CG-8 antibody (Figure 6d), in contrast, resulted in a significant increase in mean condensation number (control culture shown in Figure 6c). The condensation number in treated cultures was 1.5-fold that of the control value (Figure 6e), and the mean size of the condensations and the mean inter-condensation distance were both 0.5-fold that of control values (Figure 6f, g).

*In vivo*, CG-8 was injected into the right wings of embryos between days 5 and 6, which were then grown to days 7-8. Of 10 injected embryos, seven survived. In 3/7 cases the injection resulted in complete absence of cartilage primordia (impaired growth of humerus, radius and ulna with absence in growth of the autopod elements; Figure 6i compared to control Figure 6h), and in 4/7 cases, shortening of the zeugopod elements (Figure 6j compared to control Figure 6k)). The reduction in zeugopodial element length was on the average 40 percent of the control value (Figure 6l).

### CG-1A and CG-8 constitute a positive gene expression feedback loop

**Figure 7:**
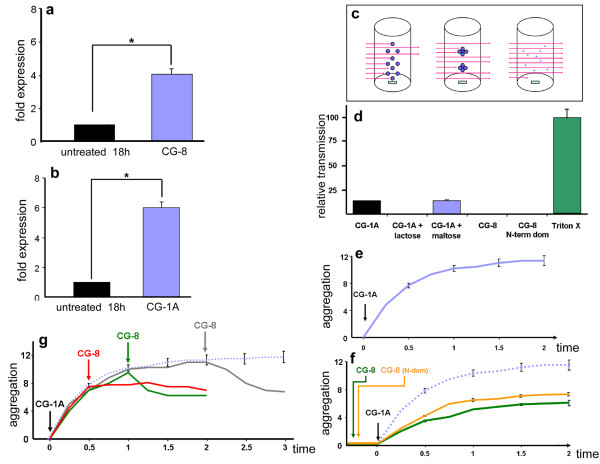
**Multiscale interactions of CG-1A and CG-8**. (a) CG-1A gene expression assayed by qRT-PCR in 18 h cultures treated with 10 *μ*g/ml CG-8 (blue bar) was 4-fold that of untreated controls (black bar). (b) CG-8 gene expression in 18 h cultures treated with 10 *μ*g/ml CG-1A (blue bar) was 6-fold that of untreated controls (black bar). Difference between relative mean mRNA values was statistically analyzed by Student's t-test. Results are shown as mean ± S.E.M. (* p < 0.05). (c) Scheme of the principle of optical aggregometry (turbidimetry). (d) Graph comparing the aggregation of 5-day leg bud autopod cells induced by addition of CG-1A, CG-1A pre-incubated with lactose or maltose (osmolarity control), and CG-8 as well as its N-terminal domain. Lysing cells with Triton X-100 (green bar) provides a baseline for maximal light transmission under the conditions of the assay. The maximum possible transmission achieved by Triton X-100 treatment is considered 100%. Results are shown as mean ± S.E.M. (e) Plot showing the cell aggregation in real time induced by CG-1A. (f) Plot showing real-time cell aggregation induced by CG-1A in cells preincubated with either CG-8 (green) or its N-terminal domain (orange). Control aggregation (dotted blue line) occurred in cells not pre-treated with CG-8. The aggregation values in assays with the N-terminal domain of CG-8 represent the mean of two independent experiments. (g) Plot showing real-time change in cell aggregation when CG-1A was added first (to initiate the aggregation) and then CG-8 was added at different time-points (red: CG-8 added at 0.5 s, green: CG-8 added at 1 s; blue: CG-8 added at 2 s). The ordinate axis units shown in e, r and g are arbitrary units and are comparable. Values for e, f and g are mean ± S.E.M.

Since galectins can affect gene expression (e.g., human galectin-1 via Sp1 transactivation [40]), we next tested for a potential effect on this level. The gene expression of CG-1A was assayed by qRT-PCR in cultures treated with 10 *μ*g/ml CG-8 at 18 hours. We found a 4-fold increase in mRNA level due to the treatment (Figure 7a). Injection of CG-8 into the autopod of 5-day wing buds in ovo also led to ectopic interdigital CG-1A RNA expression on day 6 (Additional file 3, Figure S3m) compared to uninjected counterpart wings (Additional file 3, Figure S3l). The upregulation in gene expression of CG-8 upon treatment with 10 *μ*g/ml CG-1A in 18-hour cultures was 6-fold (Figure 7b). Thus, even though CG-1A and CG-8 had opposite effects on the condensation pattern, they were actually upregulating each other's gene expression. This suggested that their effect on the pattern occurred at a level different from transcriptional regulation per se.

### CG-1A aggregates limb cells directly and CG-8 antagonizes aggregation

In general, galectins are potent adhesion/growth-regulatory effectors with cell-type selectivity, positively or negatively affecting cell-cell or cell-matrix interactions and also influencing gene expression of matrix components, as shown for human galectin-1 [41,42]. Thus, CG-1A may also affect condensation-related functions indirectly by influencing the expression, transport or degradation of cell adhesion or ECM molecules. In order to determine whether CG-1A or CG-8 presence influences condensation morphogenesis and patterning we designed an *in vitro *agglutination assay (a scheme of the working principle is shown in Figure 7c; for more details, see Materials and Methods). When 100 *μ*g CG-1A was added to a constantly stirred suspension of freshly dissociated 5-day leg autopod cells, they underwent aggregation (Figure 7d). This aggregation was inhibited by lactose but not by maltose used as osmolarity control, indicating that the precartilage cell aggregation by CG-1A is dependent on its lectin site. CG-8, or its N-terminal domain, both present in solution as monomers [29], when added in similar concentrations, did not bring about any cell aggregation. The CG-1A-induced aggregation, when traced in real time, rose sharply upon addition of the galectin and then gradually plateaued by 2 min (Figure 7e). When CG-1A was added to cells that had been pre-exposed to CG-8 or its N-terminal domain, the aggregation that resulted was less than half of that caused by CG-1A alone (Figure 7f). The presence of CG-8 thus impaired the aggregability by CG-1A. When the treatments were reversed and CG-8 was added at various points after CG-1A-dependent aggregation had begun, the aggregation ceased or dropped to about half the aggregation levels brought about by CG-1A alone (Figure 7g).

## Discussion

This paper establishes a causal association between avian limb skeletogenesis and a class of cell-cell and cell-matrix adhesion modulating gene products, the galectins. Of the three major interrelated processes in limb skeletal development, i.e., the morphogenesis of precartilage condensations [2,5], the spatiotemporal patterning of the condensations [3,43], and the specification of the identity of each skeletal element [44-46], we here demonstrate that CG-1A and CG-8 (two of the five CGs) are involved in at least the first two processes.

Classic experiments by Zwilling ([16]; later confirmed by Ros and coworkers [15]) have shown that randomized limb cells, when re-introduced under the limb ectoderm, are able to give rise to a skeleton. When cultured on plastic at high densities, dissociated early-stage limb bud cells organize themselves into regularly spaced condensations (reviewed in [3]), and later cartilage nodules, and thus replicate key events in morphogenesis occurring during limb skeletal development *in vivo *([11,13,47]; reviewed in [3]). It was therefore reasonable to assume that both the formation and spatiotemporal patterning of limb condensations is dependent on local cell-cell interactions and ensuing signaling processes [17,43,48-53]. In this context, an orchestration of expression of glycans along with counter-receptors (lectins) is an attractive concept, which guided us to the analysis of galectins, known for marked developmental regulation ([54]; see also [55] for information on glycans).

The CG-1A activity reported appears to underlie the observations in a study from nearly three decades ago [56] describing enhancement in the number of chicken limb precartilage condensations *in vitro *by a lectin from adult liver. Our initial studies of CG-1A expression, which suggested that this galectin was expressed earlier *in vitro *than fibronectin (up to now the best candidate for an ECM mediator of condensation; [11,12,47], and indeed earlier than ligands for PNA, these epitopes being among the earliest so far known molecular condensation markers [35,36], warranted pursuing detailed expression and functional studies with this and the other four CGs.

### Expression of CG-1A and CG-8 in condensing limb mesenchyme

We found that production of CG-1A and CG-8 (two out of the five CGs) mRNA and proteins were spatially and temporally associated with the formation of condensations *in vitro*. By tracing the localization of these galectins back to when they first appear within proto-condensations, we confirmed that they are indeed the earliest condensation-associated proteins reported so far.

The gene and protein expression profiles of both CGs were associated with precartilage mesenchymal condensations and early cartilage primordia *in vivo*. Similar to *in vitro *results, cells which did not participate in forming the cartilage primordia (and subsequently bone) were negative for CG-1A- or CG-8-specific mRNA and protein. Our use of an ectoderm-depleted, muscle-free limb precartilage mesenchymal population [57,58] in *in vitro *experiments permitted us to track the expression of galectins in a pure cell lineage independently of tissue interactions. Whereas no relevant data were available previously on the tandem-repeat-type CG-8, CG-1A has been analyzed immunohistochemically in embryogenesis, where it is especially strongly expressed in myogenic tissue and skin [59,60].

### Expression of galectin binding sites in condensing limb mesenchyme

Galectins, by definition, have binding affinity towards galactose-containing glycans. As with mammalian galectins, the 4'- and 6'-OH groups of galactose serve as key contact sites [61]. CG-1A preferentially targets multivalent glycans with N-acetyllactosamine termini when probed with free glycans and glycoproteins [62,63]. The observation of a gradient of decreasing binding constants during saturation of a multivalent ligand can underlie high-avidity cell aggregation even at low galectin loading [64]. The measurement of dissociation kinetics under zero-force conditions corroborated the relevance of galectin-1 in transient cell interactions [65]. Regarding natural glycoconjugates, developmental regulation of CG-1A-reactive glycans was noted in liver [66]. With respect to the biochemical nature of galectin-reactive determinants, mammalian galectins are also known to bind glycomimetic peptides and distinct motifs (e.g., in oncogenic H-ras) independent of the lectin site [25]. While elucidation of the biochemical structure of the CG-1A and CG-8 ligands in condensing leg mesenchyme was not addressed here, we assayed the spatial pattern of accessible sites by using these galectins, after activity-preserving biotinylation, as markers. The use of endogenous lectins as probes has clear advantages over glycophenotyping with plant lectins in providing information of potential functional significance. We found different patterns of localization for the accessible binding sites of CG-1A and CG-8. The binding reactivity of CG-1A was condensation-specific, its temporal expression lagged behind that of CG-1A, and was apparently subject to induction by CG-1A itself. In contrast, accessible sites for CG-8 were present both in condensing and non-condensing mesenchyme and were unaffected by CG-8 addition *in vitro*.

### Role of galectins in precartilage condensation

**Figure 8:**
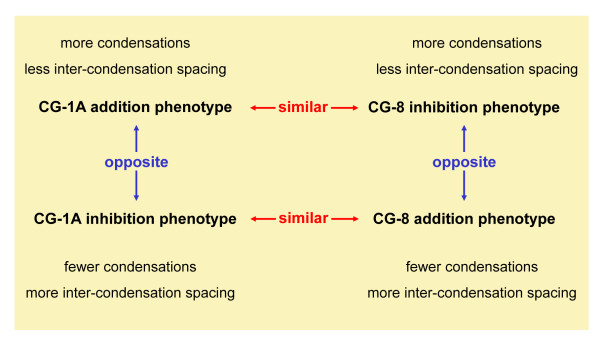
**Scheme of effects of CG-1A and -8 perturbations on condensation pattern *in vitro***.

We next examined if perturbing galectin concentrations will affect condensation morphology and pattern. We found that CG-1A and CG-8 had opposite effects on the condensation pattern: whereas addition of CG-1A increased the number and spatial density of condensations, CG-8 decreased both. Interestingly, the addition of either galectin resulted in a decrease of mean condensation size. We confirmed these effects *in vivo *as well: injection of CG-1A into the developing wing mesenchyme resulted in extra digits or fusion of existing digits. The injection of CG-8, in contrast, impaired the development and/or size of the cartilage primordia. Significantly, inhibition of the function of one galectin brought about a similar phenotype *in vitro *as the addition of the other, i.e., CG-1A inhibition was equivalent to CG-8 addition and vice versa. The relationships between the various types of treatment are shown schematically in Figure 8.

We also found in isolated pure populations of precartilage mesenchymal cells that CG-1A and CG-8 constitute a positive feedback loop at the gene expression level, i.e., they upregulate each other's expression. This finding might seem to conflict with the fact that CG-1A has an inductive effect and CG-8 an inhibitory effect on condensation formation. This apparent contradiction was resolved by probing the interaction between CG-1A and CG-8 by an aggregometry assay that provides evidence for the direct effect of galectins on limb precartilage cells. We found that CG-1A by itself was able to aggregate dissociated 5-day leg cells within two minutes. Given this rapid response, it is likely that CG-1A acts in a direct manner to mediate adhesion between cells. The developmental relevance of these rapidly forming aggregates is suggested by a recent study in which cells identical to those used here were permitted to recover from trypsinization and levitated in an ultrasound trap. Such cells formed aggregates via their native attachment moieties, also within two minutes, and contained detectable amounts of the chondrogenic transcription factor Sox9 in their nuclei within three hours [67].

CG-8 behaved differently from CG-1A in the aggregometry assay. Not only did it not aggregate cells, it also interfered with the ability of CG-1A to do so. The basis for CG-8's effect may be due to its sharing a common ligand for one of its two domains with CG-1A, thus blocking CG-1A's binding without mediating CG-1A-like cross-linking. This would be analogous to the functional divergence in post-binding events known from human galectins-1 and -3 competing for the same ligand [68,69]. Alternatively, distinct binding sites of the two galectins may be close enough on the cell surface for the non-aggregatory CG-8 to cause steric hindrance of the binding of the aggregatory CG-1A. Although it is formally also possible that CG-1A and CG-8 may interact directly to form a complex, this may require special conditions, as in solid-phase assays [70]. Given that the N-terminal domain of CG-8 (derived from the first four exons of CG-8, encoding one lectin site [29]) gives the same results in our assay as full-length CG-8, we favor the hypothesis that the N-terminus of CG-8 and CG-1A share a common ligand and compete with each other for binding.

Different ligand specificity and/or cross-linking capacity can account for non-overlapping binding profiles of CG-1A and CG-8. We suggest that the opposing roles of CG-1A and CG-8 in mediating precartilage condensation *in vitro *and *in vivo *are due to the cell-protein and protein-protein interactions reflected in the results of aggregometry assay and may occur by any of the hypothesized mechanisms. Interestingly, our preliminary evidence shows that the other CGs (i.e., CG-1B, -2 and -3), which are expressed in condensing precartilage mesenchyme (albeit to a minimal extent), are able to aggregate 5-day leg bud cells by one-third to half the extent of CG-1A. This implies that the expression and accessibility of binding sites for CGs is differentially regulated, and this in turn may regulate the ability of the CGs to cross-link cells. It also shows that the aggregability of CG-1A is not a generic property of proto-type galectins but is a product of orchestrated expression dynamics of both components of the CG-glycan recognition system.

### A dynamical schema for limb patterning based on galectins and their binding sites

We propose that the difference in regulation and spatial distribution of binding sites for CG-1A and CG-8, in concert with the lectins' properties, underlie the spatial patterning of the condensations. Local elevation of homodimeric, and thus cross-linking, CG-1A will simultaneously induce the cells to aggregate and favor further presentation of its ligand(s). This will increase the affinity of the extracellular environment within the condensations for CG-1A, effectively limiting its diffusion. CG-1A will also induce the expression of CG-8, but since CG-8 binding reactivity is uniformly distributed throughout the precondensed field of cells, CG-8, unlike CG-1A, will spread by diffusion from its sites of production (i.e., the prospective condensations). By interfering with the cell-adhesive function of CG-1A in regions peripheral to the incipient condensation, where CG-1A is sparse, CG-8 acts as a long-range lateral inhibitor of condensation formation. Since CG-1A is a local activator of condensation by also indirectly inducing its own expression via the positive feedback loop with CG-8, the two galectins may constitute the core molecules of a locally auto-activatory laterally inhibitory (LALI) network [71,72] that can mediate pattern formation of limb skeletal elements [3,73]. Computer simulations based on a partial-differential-equation representation of cells incorporating this network, using biologically plausible parameter choices, indeed exhibit quasiperiodically spaced "condensations" as well as responses to the addition of exogenous "CG-1A" similar to those seen in our experiments (Tillman Glimm, R. B. and S. A. N., in preparation).

Finally, we speculate on the possible relevance of our findings to limb development in non-avian tetrapods. All documented tetrapod species have orthologs of the avian CG-8 gene, and one or more homologs of CG-1A (ENSEMBL database, http://www.ensembl.org). In chickens, CG-1A (formerly called C-16) has a paralogous gene CG-1B (or C-14). Based on the rates of amino acid substitutions, which establish the basis for the unique redox-dependent shape changes of CG-1B [33,74], in relation to the likely evolutionary pathway, duplication of an ancestral CG-1 gene into CG-1A and -1B genes was calculated to have occurred around divergence of birds and mammals [75]. In non-avian and non-reptilian tetrapods (i.e., amphibians and mammals) the single CG-1A homolog, known as galectin-1, is actually an ortholog of CG-1B based on its chromosomal location. The *Anolis *lizard genome, like chicken, has two galectin-1-like genes, orthologs of CG-1A and CG-1B, suggesting that the duplication of an ancestral galectin-1 gene occurred after the phylogenetic split between saurians and mammals, but before evolutionary pathways of birds and lizards diverged. Significantly, using in situ hybridization with a CG-1A-specific probe, we have localized cognate gene expression in the prospective digit primordia of *Pogona vitticeps *(bearded dragon) embryos (unpublished work).

Given that galectin-1**^-/- ^**mice show no skeletal abnormalities [76], it seems unlikely that galectin-1 acts exclusively in place of CG-1A in mediating mammalian limb development. However, compensatory mechanisms in the complex galectin network have so far not been studied in KO models. The identity of the early inducer of mammalian limb mesenchymal condensations thus still needs to be determined, and our results point toward analysis of galectins. Given that mammals have at least four other proto-type galectins, any one of them with the property of specifically aggregating precartilage limb mesenchymal cells and interacting in a positive feedback expression loop with a CG-8-like protein, such as human galectin-8, a matricellular protein known to modulate cell adhesion (for a review, see [77]), could potentially substitute for CG-1A. This interplay can constitute a similar limb-patterning mechanism in mammals.

## Conclusions

We have shown that a regulatory network consisting of the galectins CG-1A and CG-8 and their respective cell surface binding moieties, operating at the levels of gene expression and protein-protein interaction, organizes avian limb bud mesenchyme into discrete skeletal elements. This network acts earlier than other known determinants or markers of skeletal pattern formation. Its ability to rapidly pattern randomized cells into regular arrangements of condensations in vitro is indicative of its self-organizing dynamics and of its independence of any limb bud signaling centers, prepatterns of transcription factors, or other early-expressed molecular gradients.

## Methods

### Chicken embryos

Fertilized White Leghorn chicken eggs were obtained from Moyer's Chicks, Quakertown, PA. Eggs were incubated in a humidified incubator at 39°C for 5-8 days, blunt end upwards for embryos used for cell cultures and horizontally for in ovo manipulations.

### Micromass cell culture

Primary cultures were prepared by pooling mesenchymal tissue dissected from the myoblast-free (distal 0.3 mm) [57,58] autopod field of leg (or in some cases wing) buds of stage 24 [78] chicken embryos (~4½ day of incubation). Cultures that contained any myoblasts, which had a distinctive bipolar morphology by 2 d, were discarded. Cells were dissociated with trypLE Express solution (Gibco, Grand Island, NY), filtered through Nytex 20 *μ*m monofilament nylon mesh (Tetko, Briarcliff Manor, NY), washed and resuspended in medium for plating at 2.5×10**^5 ^**cells per 10 *μ*l spot. Cell spots were deposited in Costar 24-well tissue culture plates (Corning Inc., Corning, NY) and allowed to attach for 1 h before the wells were flooded with 1 ml of serum-free defined medium (henceforth called DM; [79]: 60% Ham's F12, 40% Dulbecco's modified Earle's Medium (DMEM), 5 *μ*g/ml insulin, 100 nM hydrocortisone, 50 *μ*g/ml ascorbic acid, 5 *μ*g/ml chicken transferrin (Sigma, St. Louis, MO). Medium was changed daily. Under these conditions the cells grew as monolayers. Some cultures were treated with 5-20 *μ*g/ml of either CG-1A or CG-8 (in DM). In experiments where change in pattern formation was assayed, the CGs were added to freshly prepared cultures and then replaced with galectin-free media after 24 h. Inhibition (of galectin) studies used 10 *μ*g/ml of anti-CG-1A and anti-CG-8 antibodies (rabbit polyclonal; IgG). Selected cultures were stained with Alcian blue at pH 1.0 at 5 or 6 days to verify the occurrence of chondrogenesis [80].

### Quantitative real-time PCR

Total RNA was isolated from cell cultures or dissociated leg-bud cells with the Absolutely RNA Microprep kit (Stratagene, La Jolla, CA). RNA was quantified with a NanoDrop ND-1000 microspectrophotometer (NanoDrop Technologies, Rockland, DE). Per culture yields were similar for all treatment groups. Using a one-step protocol, comparative qRT-PCR was performed using a Mx3005P instrument (Stratagene) with the Brilliant II SYBR Green QRT-PCR one-step mastermix kit (Stratagene). Primers (Table 1) were designed with the Beacon Designer program (Premier Biosoft, Palo Alto, CA) and the Primerselect option of the DNASTAR Lasergene 7.1 software package (GATC Biotech, Konstanz, Germany), and evaluated using the qPCR simulation program Amplify 3× http://engels.genetics.wisc.edu/amplify/. The threshold cycle (Ct) was determined as the mean of three biological replicates by using the adaptive baseline algorithm in the MX3005P software package. All measurements were normalized to *β*-actin expression and analyzed by the ΔΔ Ct method [81].

**Table 1:** Primer sets used for qRT-PCR

Gene of interest	Accession number	Forward primer (5' to 3')	Reverse primer (5' to 3')
*β*-actin	NM205518	CGGTACCAATTACTGGTGTTAGATG	GCCTTCATTCACATCTATCACTGG

CG-1A	NM206.905.1	ATGGAGCAAGGACTGGTTGTTAC	TTAGCTGAACTTAATAGCTTTCACTTTAAAG

CG-1B	NM205.495.1	ATGGCTTGTCAGGGACC	TTACTCCCAGCTGACAGACC

CG-2	XM001234438.1	ATGGCTAGAATGTTTGAAATGTTCAACCTGG	TCACTCCACCTTGAAGGAGGTAAC

CG-3	NM214591.1	ATGTCGGACGGTTTCTCTC	TTAAATCATGGAGGTCAAAACAC

CG-8	NM001010843.1	ATGATGTCCTTGGATGGAC	CTACCAGCTCCTCACATC

The efficiencies of PCR amplification, calculated according to the method of Liu and Saint [82], averaged 0.52 for CG-1A (range: 0.49-0.54), 0.51 for CG-1B (0.50-0.54), 0.43 for CG-2 (0.39-0.48) and 0.51 for CG-8 (0.47-0.60). We therefore estimate that the PCR amplification efficiencies for CG-1A and CG-8 are comparable, within a factor of 1.02 of each other and within factors of 1.007 and 1.21 for CG-1B and CG-2, respectively.

### CG production, labeling and antibody generation

CG-1A, full-length CG-8 and its N-terminal domain were obtained by recombinant production, purified by affinity chromatography as central step and checked for purity by one- and two-dimensional gel electrophoresis [29,63,83]. The proteins were used as antigens to generate polyclonal antibodies in rabbits, and resulting affinity-purified immunoglobulin G preparations were routinely checked for lack of cross-reactivity to the other CGs systematically, including complete removal of any such reactivity by a further affinity chromatography on CG-loaded resins [84]. Biotin labeling was performed with 0.5 mg lectin (in 2 ml 2 mM phosphate-buffered saline at pH 8.0 containing 20 mM lactose to protect amino acid side chains required for ligand binding for 14 h at 4°C using 3 mg biotinyl-N-hydroxysuccinimide ester (Sigma, Munich, Germany) dissolved in dimethylformamide, the extent of biotin incorporation determined by mass spectrometry and the carbohydrate-dependent binding activity of the biotinylated CGs ascertained by solid-phase and cell assays [29,85].

### Immunostaining and signal ratioing

Limb mesenchymal cultures were fixed for 20 min with 100% methanol precooled to -20°C, washed three times for 5 min with PBS and permeabilized with 0.02% Triton X-100 for 10 min. Very early (6-9 h) cultures were fixed with Bouin's solution, having yielded optimal signal-to-noise ratio and antigen reactivity in comparative analysis on tissue sections [31,86]. Immunohistochemistry was performed on fixed cultures using Histostain-Plus kit (Zymed Inc., San Francisco, CA) according to the manufacturer's protocol. Briefly, the cultures were incubated with 5 mg/ml affinity-purified polyclonal rabbit anti-CG-1A or anti-CG-8 antibodies for 2 h at room temperature. Following removal of unbound primary antibody by repeated PBS washes the cultures were successively incubated for 10 min each with solutions containing a biotinylated broad-spectrum secondary antibody, the streptavidin-peroxidase conjugate and a 1:2:1 combination of 0.6% hydrogen peroxide, diaminobenzidine (DAB) substrate solution and a substrate buffer. Finally, cultures were then rinsed with water and photographed under a binocular dissecting microscope at 1.2× magnification to visualize the staining pattern of the whole micromass culture or through an inverted microscope at 16× or 32× objective magnification to visualize selected fields. Controls for antigen dependence of staining were incubated without primary antibodies, or secondary antibodies. Another set of control cultures were incubated with primary antibodies pre-incubated with the respective galectins for saturation.

To verify the condensation-specific expression of galectins, cultures were stained for CG-1A (or CG-8) and for cell nuclei using DAPI. The border between a single condensation and its surrounding uncondensed mesenchyme was photographed using a high-magnification objective (63× oil immersion lens) under filters that allowed the visualization of DAPI fluorescence and that of the fluorophore DyLight 594 conjugated to the secondary goat anti-rabbit antibody. After grayscaling the images, the mean fluorescence intensity was measured in three different but equal-sized areas (encompassing ~3 cells) inside and outside condensations, and intensity values of the condensed cells were compared to those of the non-condensed cells. Quantitative comparison to analogous values obtained for DAPI resulted in normalized values for the density-dependent differential fluorescence in the cultures.

For immunostaining of tissue sections, whole embryos were fixed overnight in 4% paraformaldehyde. 10 *μ*m paraffin sections were deparaffinized, rehydrated, treated for antigen retrieval by microwaving for 5 min in a bath of 1% zinc sulfate solution [87], and incubated in blocking solution (from Histostain-plus kit; Zymed Laboratories) prior to incubation with primary anti-galectin antibodies at room temperature for 1 hour. Antigen detection was performed using the Histostain-Plus kit following the manufacturer's protocol.

### Lectin histochemistry

Biotinylated CG-1A and -8 were used to visualize the spatial distribution of accessible sites reactive with CG-1A and CG-8 *in vitro *and in leg sections. Using a protocol similar to that described above for indirect immunohistochemistry and tested for a mammalian galectin including specificity controls [88], fixed mesenchymal cultures were incubated with 5 *μ*g/ml biotinylated CG-1A or CG-8 at 4°C overnight. This was followed by treatment with the streptavidin-peroxidase conjugate and chromogenic substrates as described above. Control cultures were treated with biotinylated CGs that had been pre-incubated with lactose. Peanut agglutinin staining was performed on cultures fixed on days 2 or 3. Cultures were incubated with 50 *μ*g/ml horseradish-peroxidase (HRP)-conjugated PNA (Sigma, St. Louis, MO) for 1 h followed by DAB color reaction. In fluorescence colocalization experiments, cultures were incubated at room temperature for 1 h with 50 *μ*g/ml FITC-conjugated PNA (Sigma).

### Whole mount in situ hybridization

In situ hybridization was performed on whole embryos essentially as described [89]. Clone 1A/B and Clone 15/1, containing inserts (405 base pairs for CG-1A and 948 base pairs for CG-8) into the pGEM-4Z vector (Promega, Mannheim, Germany), were linearized with HindIII and EcoRI, for synthesis of labeled antisense strands SP6 polymerase and T7 polymerase, respectively, and the digoxigenin-based DIG RNA labeling kit were used (Roche, Indianapolis, IN). Probe synthesis was carried out for 2 h at 37°C, followed by a 15 min incubation at 37°C with 2 *μ*l of RNase-free DNase I to remove the DNA template. Probe concentration was determined by absorbance at 260 nm. Probes were evaluated for digoxigenin incorporation by slot blotting using the BCIP/NBT liquid substrate detection system (Sigma, St. Louis, MO). All the prehybridization and post hybridization washes were performed using the BioLane HTI 16V tissue processing robot (Intavis Bioanytical Instruments, Koeln, Germany).

### In ovo manipulation of wing buds and cartilage staining

Chicken eggs were candled on the third day of incubation to locate the position of the embryos and 3-4 ml of albumin was removed [90]. Between 5-6 days ~1 *μ*g of CG-1A or CG-8 (2 *μ*g/ml in nuclease-free PBS) was injected into the mesenchyme of the autopod field of the wing bud. Control embryos were injected with identical amounts of BSA. The egg was resealed and the embryos were allowed to grow for 2-3 more days. The use of wing buds was preferred to leg buds due to access. The sites of galectin injections included the interdigit mesenchyme and digit primordia. Embryos at 7½ - 8½ days of incubation were washed in PBS, fixed in 5% trichloroacetic acid overnight, and then stained overnight in 0.1% Alcian blue in acid alcohol. Following overnight destaining in acid alcohol, embryos were dehydrated in absolute ethanol and cleared in methyl salicylate.

### Knockdown of CG-1A

In order to inhibit production of CG-1A within limb mesenchymal cultures, siRNA oligonucleotide targeting the sequence (5'-GAGAGTGTGTCAAGGTCAA-3') corresponding to nucleotides 44-62 of the CG-1A open reading frame and the scrambled control were designed using the on-line siRNA design tool on http://Invitrogen.com. For each culture, 2 *μ*g of oligonucleotides incubated for 20 min with 10 *μ*l of Metafectene Pro (Biontex Laboratories, Munich, Germany) and added dropwise to the cultures. The cultures were harvested and checked for knockdown by assaying CG-1A-specific levels by qRT-PCR and CG-1A protein levels by indirect immunofluorescence. For injections, 2 *μ*g of oligonucleotides and 2 *μ*l of Lipofectamine 2000 (Invitrogen, Carlsbad, CA) were both dissolved in 30 *μ*l of nuclease free water (Fisher Scientific, Pittsburgh, PA), mixed and incubated for 20 mins. Each 5-day wing bud was injected with 1.5-2 *μ*l of the mixture.

### Limb cell turbidimetry

The capacity of galectins to directly mediate cell-cell adhesion was assayed by a turbidimetric method adapted from platelet clotting studies [91,92]. Briefly, cells were dispersed uniformly in DM inside a cuvette. Light transmitted through the cuvette is detected by a sensor. The suspended particles (e.g., cells) scatter light, but the turbidity is reduced if the cells are caused to aggregate. The effect of galectins on precartilage mesenchymal cells was assayed using a Chrono-Log lumi-aggregometer model 600 (Havertown, PA). The cell density was 2.5 × 10^6 ^per ml and the concentration of CG-1A and CG-8 (or its N-terminal domain) used was 10 *μ*g/ml. In control experiments the suspension was made 5 mM in lactose or maltose. Maximal light transmission resulting from aggregation in this experimental setup was determined by lysing the cells with Triton X-100, allowing the light to pass through with negligible scattering.

### Statistics of pattern formation

The effect of CGs, their antibodies and CG-1A-specific siRNA on the *in vitro *condensation pattern was assessed quantitatively in terms of three variables: condensation size, condensation number and intercondensation distance. Control and treated cultures were grown for 2 days, fixed with absolute methanol and stained with DAPI. Concentrations of galectins and their antibodies) were chosen for the condensation pattern-perturbing experiments by trial and error to ensure the diameters of the treated micromasses at 2 days did not differ from untreated controls. Mammalian galectins 1 and 8 have been shown to be pro-apoptotic [93]. While we have seen this effect with CG-8 (but not CG-1A [38]), the concentrations of the CGs used in the experiments described here were only 20% of the pro-apoptotic levels of CG-8.

The cultures were photographed under low magnification (2.5×) and the images were binarized using ImageJ software http://rsb.info.nih.gov/ij/. The smallest condensation was chosen by eye from among all the culture pictures and was set as the threshold for the lower limit of condensation size measurement. Image J was then used to automatically measure the number and sizes of condensations in each picture.

For measurement of inter-condensation distances, an arbitrary point was chosen on the boundary of each condensation. This point was then connected with the point on the boundary of the condensation that was farthest from it, such that the line connecting the two points lay within the condensation. Since most of the condensations were quasi-circular, the line joining the two points represented an approximation to the diameter of the condensation. The mid-point of this axis was taken to be the center of the condensation. The centers of the condensations were then connected to those of neighboring condensations and the inter-condensation distances were measured. In cultures which were immunostained using a color reaction, the whole micromasses were photographed and the resulting images were then grayscaled and segmented into stacks using multiOtsu thresholding [94].

The values of the condensation numbers, sizes and inter-condensation distances were represented as fold means +/- S.E.M. The statistical significance of the difference between groups and controls was assessed by one-way analysis of variance with Tukey-Kramer post-hoc analysis. The difference was taken to be significant if the p-value was < 0.05.

## Authors' contributions

RB carried out the in vitro and embryological manipulations, the quantitative RT-PCR assays and the in situ hybridizations. RB and KML performed the aggregometric assays, and RB and HP the histochemical assays. HK prepared and characterized the galectins and galectin-related reagents. H-JG and SAN conceived of the study and, with RB and KML designed the experiments. SAN, RB and H-JG wrote the manuscript, which was read and approved by all the authors.

## Supplementary Material

Additional file 1 **Figure S1: CG-1A and -8 protein expression *in vitro***. (a-b) Spatial localization of CG-8 by indirect immunostaining in 3-day micromass leg bud mesenchymal cultures shown at low magnification (a) and high magnification (b). (c) Spatial localization of CG-8 by indirect immunostaining in 9-hour fixed cultures shows its moderately specific presence within proto-condensations (black arrowheads). (d) Graph showing condensation-specific fluorescence for CG-1A relative to DAPI fluorescence in non-condensed cells. (e) Graph showing condensation-specific fluorescence for CG-8 relative to DAPI fluorescence in non-condensed cells. Values for (d) and (e) are mean ± S.E.M. (* p < 0.05). Click here for file

Additional file 2 **Figure S2: Expression of CG-8 in developing chick leg buds**. (a-b) Spatial expression of CG-8-specific mRNA in (a) 5-day and (b) 6-day leg buds by in situ hybridization. (c-d) Staining for CG-8 in the prospective stylopod area of a (c) 3½-day and (d) 5-day chick leg bud section, visualized by immunohistochemistry with hematoxylin counterstain. (e) Staining for CG-8 protein in 6-day leg bud section (only autopod and distal edge of zeugopodial primordia shown). The leading edge of the fourth digit primordia (outlined by the red box) shows strong crescent-shaped CG-8-specific staining (red arrowhead) at its distal edge with weak staining in cells both inside the primordia and outside. The CG-8-specific staining within the crescent fills up the extracellular space (outlined by the yellow box) whereas the extracellular space in the interdigit area is clear of CG-8 staining (outlined by blue box). The pull-out detail image in red box was photographed with 10× objective and the scale bar represents 0.2 mm. The detail images enclosed by yellow and blue boxes were photographed with 63 × oil immersion objective and the scale bar in the blue box represents 50 *μ*m. Click here for file

Additional file 3 **Figure S3: Knockdown of CG-1A expression by RNAi decreases condensation number and size**. (a) Indirect immunolocalization of CG-1A in a 2-day control leg culture. (b, c) Treatment of 2-day leg cultures with (b) CG-1A siRNA and (c) scrambled control oligo, both stained for CG-1A protein. (d) Relative CG-1A expression measured by qRT-PCR in untreated control, upon treatment with the scrambled control oligonucleotide, and with CG-1A siRNA. Analysis was done using Student's t-test. Results are shown as mean ± S.E.M. (*p < 0.05). (e) Control 2-day culture stained with DAPI. (f) Culture treated with CG-1A-targeting siRNA, fixed at day 2, and stained with DAPI. Photomicrographs (a-c) are at same level of magnification; bar in (a) represents 0.2 mm. Photomicrographs (d) and (e) are at same magnification and the bar in (d) represents 0.25 mm. (g) Graph showing change in mean condensation number upon knockdown of CG-1A-specific mRNA by RNAi (number in untreated control shown by black bar). (h) Graph showing change in mean condensation size upon knockdown of CG-1A-specific mRNA by RNAi. (i) Graph showing lack of significant alteration in mean inter-condensation distance upon treatment with CG-1A-targeting siRNA. The three variables (condensation number, size and inter-condensation distance) were analyzed as in the legend to Figure 5. (j, k) Untreated 7-day control wing stained with Alcian blue showing two digit primordia (j), whereas injection of CG-1A siRNA-Lipofectamine mixture into the counterpart wing on the 5^th ^day of incubation resulted in the autopodium lacking any cartilage (k). (l, m) Untreated 6-day control wing stained for CG-1A mRNA shows a digit-like pattern in the autopodium (l) whereas injection of CG-8 in the counterpart wing on the 5^th ^day of incubation leads to increased spatial localization and stronger CG-1A mRNA staining (m). Click here for file

## References

[B1] Gilbert SF Developmental biology 2010 9 Sunderland, MA: Sinauer Associates

[B2] Hall BK Miyake T Divide, accumulate, differentiate: cell condensation in skeletal development revisited Int J Dev Biol 1995 39 881 893 8901191

[B3] Newman SA Bhat R Activator-inhibitor dynamics of vertebrate limb pattern formation Birth Defects Res C Embryo Today 2007 81 305 319 10.1002/bdrc.20112 18228262

[B4] Fell HB Canti RG Experiments on the development in vitro of the avian knee-joint Proc R Soc Lond B 1934 116 316 351 10.1098/rspb.1934.0076

[B5] Hall BK Miyake T All for one and one for all: condensations and the initiation of skeletal development Bioessays 2000 22 138 147 10.1002/(SICI)1521-1878(200002)22:2<138::AID-BIES5>3.0.CO;2-4 10655033

[B6] Newman SA Christley S Glimm T Hentschel HG Kazmierczak B Zhang YT Zhu J Alber M Multiscale models for vertebrate limb development Curr Top Dev Biol 2008 81 311 340 full_text 18023733 10.1016/S0070-2153(07)81011-8

[B7] Saga Y Yagi T Ikawa Y Sakakura T Aizawa S Mice develop normally without tenascin Genes Dev 1992 6 1821 1831 10.1101/gad.6.10.1821 1383086

[B8] Cremer H Lange R Christoph A Plomann M Vopper G Roes J Brown R Baldwin S Kraemer P Scheff S Barthels D Rajewsky K Wille W Inactivation of the N-CAM gene in mice results in size reduction of the olfactory bulb and deficits in spatial learning Nature 1994 367 455 459 10.1038/367455a0 8107803

[B9] Luo Y Kostetskii I Radice GL N-cadherin is not essential for limb mesenchymal chondrogenesis Dev Dyn 2005 232 336 344 10.1002/dvdy.20241 15614770

[B10] Downie SA Newman SA Morphogenetic differences between fore and hind limb precartilage mesenchyme: relation to mechanisms of skeletal pattern formation Dev Biol 1994 162 195 208 10.1006/dbio.1994.1078 8125187

[B11] Frenz DA Jaikaria NS Newman SA The mechanism of precartilage mesenchymal condensation: a major role for interaction of the cell surface with the amino-terminal heparin-binding domain of fibronectin Dev Biol 1989 136 97 103 10.1016/0012-1606(89)90133-4 2806726

[B12] Gehris AL Stringa E Spina J Desmond ME Tuan RS Bennett VD The region encoded by the alternatively spliced exon IIIA in mesenchymal fibronectin appears essential for chondrogenesis at the level of cellular condensation Dev Biol 1997 190 191 205 10.1006/dbio.1997.8693 9344538

[B13] Moftah MZ Downie SA Bronstein NB Mezentseva N Pu J Maher PA Newman SA Ectodermal FGFs induce perinodular inhibition of limb chondrogenesis in vitro and in vivo via FGF receptor 2 Dev Biol 2002 249 270 282 10.1006/dbio.2002.0766 12221006

[B14] Zeller R Lopez-Rios J Zuniga A Vertebrate limb bud development: moving towards integrative analysis of organogenesis Nat Rev Genet 2009 10 845 858 10.1038/nrg2681 19920852

[B15] Ros MA Lyons GE Mackem S Fallon JF Recombinant limbs as a model to study homeobox gene regulation during limb development Dev Biol 1994 166 59 72 10.1006/dbio.1994.1296 7958460

[B16] Zwilling E Development of fragmented and of dissociated limb bud mesoderm Dev Biol 1964 89 20 37 10.1016/0012-1606(64)90012-0 14118783

[B17] Leonard CM Fuld HM Frenz DA Downie SA Massagué J Newman SA Role of transforming growth factor-β in chondrogenic pattern formation in the embryonic limb: stimulation of mesenchymal condensation and fibronectin gene expression by exogenous TGF-β and evidence for endogenous TGF-β-like activity Dev Biol 1991 145 99 109 10.1016/0012-1606(91)90216-P 2019328

[B18] Montero JA Lorda-Diez CI Gañan Y Macias D Hurle JM Activin/TGFβ and BMP crosstalk determines digit chondrogenesis Dev Biol 2008 321 343 356 10.1016/j.ydbio.2008.06.022 18602912

[B19] Saunders JW Jr The proximo-distal sequence of origin of the parts of the chick wing and the role of the ectoderm J Exp Zool 1948 108 363 402 10.1002/jez.1401080304 18882505

[B20] Villalobo A Nogales-Gonzalés A Gabius H-J A guide to signaling pathways connecting protein-glycan interaction with the emerging versatile effector functionality of mammalian lectins Trends Glycosci Glycotechnol 2006 18 1 37

[B21] André S Sanchez-Ruderisch H Nakagawa H Buchholz M Kopitz J Forberich P Kemmner W Böck C Deguchi K Detjen KM Tumor suppressor p16INK4a--modulator of glycomic profile and galectin-1 expression to increase susceptibility to carbohydrate-dependent induction of anoikis in pancreatic carcinoma cells FEBS J 2007 274 3233 3256 17535296 10.1111/j.1742-4658.2007.05851.x

[B22] Roda O Ortiz-Zapater E Martinez-Bosch N Gutiérrez-Gallego R Vila-Perello M Ampurdanés C Gabius H-J André S Andreu D Real FX Navarro P Galectin-1 is a novel functional receptor for tissue plasminogen activator in pancreatic cancer Gastroenterology 2009 136 e1371 1375 1379-1390 10.1053/j.gastro.2008.12.039 19171142

[B23] Wang J Lu ZH Gabius H-J Rohowsky-Kochan C Ledeen RW Wu G Cross-linking of GM1 ganglioside by galectin-1 mediates regulatory T cell activity involving TRPC5 channel activation: possible role in suppressing experimental autoimmune encephalomyelitis J Immunol 2009 182 4036 4045 10.4049/jimmunol.0802981 19299701

[B24] Gabius HJ The sugar code: fundamentals of glycosciences 2009 Weinheim Chichester: Wiley-VCH

[B25] Gabius H-J Cell surface glycans: the why and how of their functionality as biochemical signals in lectin-mediated information transfer Crit Rev Immunol 2006 26 43 79 16472068 10.1615/critrevimmunol.v26.i1.30

[B26] Kopitz J Bergmann M Gabius H-J How adhesion/growth-regulatory galectins-1 and -3 attain cell specificity: Case study defining their target on neuroblastoma cells (SK-N-MC) and marked affinity regulation by affecting microdomain organization of the membrane IUBMB Life 2010 62 624 628 10.1002/iub.358 20665623

[B27] Shoji H Nishi N Hirashima M Nakamura T Characterization of the Xenopus galectin family. Three structurally different types as in mammals and regulated expression during embryogenesis J Biol Chem 2003 278 12285 12293 10.1074/jbc.M209008200 12538594

[B28] Gabius H-J Gabius H-J Animal and human lectins The Sugar Code Fundamentals of glycosciences 2009 Weinheim: Wiley-VCH 317 328

[B29] Kaltner H Solis D André S Lensch M Manning JC Mürnseer M Sáiz JL Gabius H-J Unique chicken tandem-repeat-type galectin: implications of alternative splicing and a distinct expression profile compared to those of the three proto-type proteins Biochemistry 2009 48 4403 4416 10.1021/bi900083q 19344160

[B30] Cooper DN Galectinomics: finding themes in complexity Biochim Biophys Acta 2002 1572 209 231 12223271 10.1016/s0304-4165(02)00310-0

[B31] Kaltner H Solis D Kopitz J Lensch M Lohr M Manning JC Murnseer M Schnölzer M André S Sáiz JL Gabius H-J Prototype chicken galectins revisited: characterization of a third protein with distinctive hydrodynamic behaviour and expression pattern in organs of adult animals Biochem J 2008 409 591 599 10.1042/BJ20070419 17887955

[B32] Kaltner H Kübler D López-Merino L Lohr M Manning JC Lensch M Seidler J Lehmann WD André S Solís D Gabius H-J Towards comprehensive analysis of the galectin network in chicken: unique diversity of galectin-3 and comparison of its localization profile in organs of adult animals to the other four members of this lectin family Anat Rec in press 10.1002/ar.21341 21290613

[B33] Varela PF Solis D Diaz-Mauriño T Kaltner H Gabius H-J Romero A The 2.15 A crystal structure of CG-16, the developmentally regulated homodimeric chicken galectin J Mol Biol 1999 294 537 549 10.1006/jmbi.1999.3273 10610778

[B34] Beyer EC Zweig SE Barondes SH Two lactose binding lectins from chicken tissues. Purified lectin from intestine is different from those in liver and muscle J Biol Chem 1980 255 4236 4239 6768752

[B35] Aulthouse AL Solursh M The detection of a precartilage, blastema-specific marker Dev Biol 1987 120 377 384 10.1016/0012-1606(87)90240-5 3556759

[B36] Milaire J Lectin binding sites in developing mouse limb buds Anat Embryol (Berl) 1991 184 479 488 10.1007/BF01236054 1741479

[B37] Burke AC Feduccia A Developmental patterns and the identification of homologies in the avian hand Science 1997 278 666 668 10.1126/science.278.5338.666

[B38] Bhat R Molecular and dynamical mediators of avian limb pattern formation 2010 Ph.D. thesis New York Medical College, Valhalla, NY

[B39] Hurle JM Colombatti A Extracellular matrix modifications in the interdigital spaces of the chick embryo leg bud during the formation of ectopic digits Anat Embryol (Berl) 1996 193 355 364 10.1007/BF00186692 8694271

[B40] Fischer C Sanchez-Ruderisch H Welzel M Wiedenmann B Sakai T André S Gabius H-J Khachigian L Detjen KM Rosewicz S Galectin-1 interacts with the α5β1 fibronectin receptor to restrict carcinoma cell growth via induction of p21 and p27 J Biol Chem 2005 280 37266 37277 10.1074/jbc.M411580200 16105842

[B41] André S Kojima S Yamazaki N Fink C Kaltner H Kayser K Gabius H-J Galectins-1 and -3 and their ligands in tumor biology. Non-uniform properties in cell-surface presentation and modulation of adhesion to matrix glycoproteins for various tumor cell lines, in biodistribution of free and liposome-bound galectins and in their expression by breast and colorectal carcinomas with/without metastatic propensity J Cancer Res Clin Oncol 1999 125 461 474 10480338 10.1007/s004320050303 PMC12172401

[B42] Camby I Decaestecker C Lefranc F Kaltner H Gabius H-J Kiss R Galectin-1 knocking down in human U87 glioblastoma cells alters their gene expression pattern Biochem Biophys Res Commun 2005 335 27 35 10.1016/j.bbrc.2005.07.037 16051185

[B43] Yang Y Growth and patterning in the limb: signaling gradients make the decision Sci Signal 2009 2 pe3 10.1126/scisignal.253pe3 19141858

[B44] Vargas AO Fallon JF The digits of the wing of birds are 1, 2, and 3. A review J Exp Zool B Mol Dev Evol 2005 304 206 219 10.1002/jez.b.21051 15880771

[B45] Scherz PJ McGlinn E Nissim S Tabin CJ Extended exposure to Sonic hedgehog is required for patterning the posterior digits of the vertebrate limb Dev Biol 2007 308 343 354 10.1016/j.ydbio.2007.05.030 17610861 PMC2100419

[B46] Zhu J Nakamura E Nguyen MT Bao X Akiyama H Mackem S Uncoupling Sonic hedgehog control of pattern and expansion of the developing limb bud Dev Cell 2008 14 624 632 10.1016/j.devcel.2008.01.008 18410737 PMC8284562

[B47] Frenz DA Akiyama SK Paulsen DF Newman SA Latex beads as probes of cell surface-extracellular matrix interactions during chondrogenesis: evidence for a role for amino-terminal heparin-binding domain of fibronectin Dev Biol 1989 136 87 96 10.1016/0012-1606(89)90132-2 2509263

[B48] Newman SA Frisch HL Dynamics of skeletal pattern formation in developing chick limb Science 1979 205 662 668 10.1126/science.462174 462174

[B49] Merino R Gañan Y Macias D Economides AN Sampath KT Hurle JM Morphogenesis of digits in the avian limb is controlled by FGFs, TGFβs, and noggin through BMP signaling Dev Biol 1998 200 35 45 10.1006/dbio.1998.8946 9698454

[B50] Miura T Shiota K TGFβ2 acts as an "activator" molecule in reaction-diffusion model and is involved in cell sorting phenomenon in mouse limb micromass culture Dev Dyn 2000 217 241 249 10.1002/(SICI)1097-0177(200003)217:3<241::AID-DVDY2>3.0.CO;2-K 10741418

[B51] Kingsley DM Genetic control of bone and joint formation Novartis Found Symp 2001 232 213 222 discussion 222-234, 272-282full_text 11277082 10.1002/0470846658.ch15

[B52] Mariani FV Martin GR Deciphering skeletal patterning: clues from the limb Nature 2003 423 319 325 10.1038/nature01655 12748649

[B53] Tickle C Making digit patterns in the vertebrate limb Nat Rev Mol Cell Biol 2006 7 45 53 10.1038/nrm1830 16493412

[B54] Barondes SH Soluble lectins: a new class of extracellular proteins Science 1984 223 1259 1264 10.1126/science.6367039 6367039

[B55] Habermann FA Sinowatz F Gabius H-J Glycobiology of fertilization and early embryonic development The Sugar Code Fundamentals of glycosciences 2009 Weinheim: Wiley-VCH 403 417

[B56] Matsutani E Yamagata T Chick endogenous lectin enhances chondrogenesis of cultured chick limb bud cells Dev Biol 1982 92 544 548 10.1016/0012-1606(82)90199-3 7117700

[B57] Newman SA Pautou M-P Kieny M The distal boundary of myogenic primordia in chimeric avian limb buds and its relation to an accessible population of cartilage progenitor cells Dev Biol 1981 84 440 448 10.1016/0012-1606(81)90413-9 10694927

[B58] Brand B Christ B Jacob HJ An experimental analysis of the developmental capacities of distal parts of avian leg buds Am J Anat 1985 173 321 340 10.1002/aja.1001730408 20726129

[B59] Levi G Teichberg VI Patterns of expression of a 15 K beta-D-galactoside-specific lectin during early development of the avian embryo Development 1989 107 909 921 12356092 10.1242/dev.107.4.909

[B60] Akimoto Y Obinata A Hirabayashi J Sakakura Y Endo H Kasai K Hirano H Changes in expression of two endogenous beta-galactoside-binding isolectins in the dermis of chick embryonic skin during development in ovo and in vitro Cell Tissue Res 1995 279 3 12 10.1007/BF00300686 7895262

[B61] Solis D Romero A Kaltner H Gabius H-J Diaz-Mauriño T Different architecture of the combining site of the two chicken galectins revealed by chemical mapping studies with synthetic ligand derivatives J Biol Chem 1996 271 12744 12748 10.1074/jbc.271.22.12744 8662681

[B62] Wu AM Singh T Liu JH Krzeminski M Russwurm R Siebert HC Bonvin AM André S Gabius H-J Activity-structure correlations in divergent lectin evolution: fine specificity of chicken galectin CG-14 and computational analysis of flexible ligand docking for CG-14 and the closely related CG-16 Glycobiology 2007 17 165 184 10.1093/glycob/cwl062 17060369

[B63] Wu AM Wu JH Tsai MS Kaltner H Gabius H-J Carbohydrate specificity of a galectin from chicken liver (CG-16) Biochem J 2001 358 529 538 10.1042/0264-6021:3580529 11535116 PMC1222089

[B64] Dam TK Gabius H-J André S Kaltner H Lensch M Brewer CF Galectins bind to the multivalent glycoprotein asialofetuin with enhanced affinities and a gradient of decreasing binding constants Biochemistry 2005 44 12564 12571 10.1021/bi051144z 16156668

[B65] Dettmann W Grandbois M André S Benoit M Wehle AK Kaltner H Gabius H-J Gaub HE Differences in zero-force and force-driven kinetics of ligand dissociation from beta-galactoside-specific proteins (plant and animal lectins, immunoglobulin G) monitored by plasmon resonance and dynamic single molecule force microscopy Arch Biochem Biophys 2000 383 157 170 10.1006/abbi.2000.1993 11185549

[B66] Lips KS Kaltner H Reuter G Stierstorfer B Sinowatz F Gabius H-J Correspondence of gradual developmental increases of expression of galectin-reactive glycoconjugates with alterations of the total contents of the two differentially regulated galectins in chicken intestine and liver as indication for overlapping functions Histol Histopathol 1999 14 743 760 10425543 10.14670/HH-14.743

[B67] Edwards GO Coakley WT Ralphs JR Archer CW Modelling condensation and the initiation of chondrogenesis in chick wing bud mesenchymal cells levitated in an ultrasound trap Eur Cell Mater 2010 19 1 12 20077400 10.22203/ecm.v019a01

[B68] Kopitz J von Reitzenstein C André S Kaltner H Uhl J Ehemann V Cantz M Gabius H-J Negative regulation of neuroblastoma cell growth by carbohydrate-dependent surface binding of galectin-1 and functional divergence from galectin-3 J Biol Chem 2001 276 35917 35923 10.1074/jbc.M105135200 11451961

[B69] Sanchez-Ruderisch H Fischer C Detjen K Welzel M Wimmel A Manning JC André S Gabius H-J Tumor suppressor p16^INK4a^: downregulation of galectin-3, an endogenous competitor of the pro-anoikis effector galectin-1, in a pancreatic carcinoma model FEBS J 2010 277 3552 3563 10.1111/j.1742-4658.2010.07764.x 20695889

[B70] Solis D Maté MJ Lohr M Ribeiro JP Lopez-Merino L André S Buzamet E Cañada FJ Kaltner H Lensch M N-domain of human adhesion/growth-regulatory galectin-9: preference for distinct conformers and non-sialylated N-glycans and detection of ligand-induced structural changes in crystal and solution Int J Biochem Cell Biol 2010 42 1019 1029 10.1016/j.biocel.2010.03.007 20227520

[B71] Meinhardt H Gierer A Pattern formation by local self-activation and lateral inhibition BioEssays 2000 22 753 760 10.1002/1521-1878(200008)22:8<753::AID-BIES9>3.0.CO;2-Z 10918306

[B72] Nijhout HF Müller GB, Newman SA Gradients, diffusion and genes in pattern formation Origination of Organismal Form: Beyond the Gene in Developmental and Evolutionary Biology 2003 Cambridge, MA.: MIT Press 165 181

[B73] Zhu J Zhang YT Alber MS Newman SA Bare bones pattern formation: a core regulatory network in varying geometries reproduces major features of vertebrate limb development and evolution PLoS One 2010 5 e10892 10.1371/journal.pone.0010892 20531940 PMC2878345

[B74] López-Lucendo MF Solis D Sáiz JL Kaltner H Russwurm R André S Gabius H-J Romero A Homodimeric chicken galectin CG-1B (C-14): Crystal structure and detection of unique redox-dependent shape changes involving inter- and intrasubunit disulfide bridges by gel filtration, ultracentrifugation, site-directed mutagenesis, and peptide mass fingerprinting J Mol Biol 2009 386 366 378 18848566 10.1016/j.jmb.2008.09.054

[B75] Sakakura Y Hirabayashi J Oda Y Ohyama Y Kasai K Structure of chicken 16-kDa beta-galactoside-binding lectin. Complete amino acid sequence, cloning of cDNA, and production of recombinant lectin J Biol Chem 1990 265 21573 21579 2254315

[B76] Poirier F Robertson EJ Normal development of mice carrying a null mutation in the gene encoding the L14 S-type lectin Development 1993 119 1229 1236 8306885 10.1242/dev.119.4.1229

[B77] Cludts S Decaestecker C Mahillon V Chevalier D Kaltner H André S Remmelink M Leroy X Gabius H-J Saussez S Galectin-8 up-regulation during hypopharyngeal and laryngeal tumor progression and comparison with galectin-1, -3 and -7 Anticancer Res 2009 29 4933 4940 20044599

[B78] Hamburger V Hamilton HL A series of normal stages in the development of the chick embryo J Morphol 1951 88 49 92 10.1002/jmor.1050880104 24539719

[B79] Paulsen DF Solursh M Microtiter micromass cultures of limb-bud mesenchymal cells In Vitro Cell Dev Biol 1988 24 138 147 10.1007/BF02623891 3343192

[B80] Lev R Spicer SS Specific staining of sulfate groups with alcian blue at low pH J Histochem Cytochem 1964 12 309 14187344 10.1177/12.4.309

[B81] Livak KJ Schmittgen TD Analysis of relative gene expression data using real-time quantitative PCR and the 2(-Delta Delta C(T)) Method Methods 2001 25 402 408 10.1006/meth.2001.1262 11846609

[B82] Liu W Saint DA A new quantitative method of real time reverse transcription polymerase chain reaction assay based on simulation of polymerase chain reaction kinetics Anal Biochem 2002 302 52 59 10.1006/abio.2001.5530 11846375

[B83] Sarter K André S Kaltner H Lensch M Schulze C Urbonaviciute V Schett G Herrmann M Gabius H-J Detection and chromatographic removal of lipopolysaccharide in preparations of multifunctional galectins Biochem Biophys Res Commun 2009 379 155 159 10.1016/j.bbrc.2008.12.024 19101505

[B84] Lensch M Lohr M Russwurm R Vidal M Kaltner H André S Gabius H-J Unique sequence and expression profiles of rat galectins-5 and -9 as a result of species-specific gene divergence Int J Biochem Cell Biol 2006 38 1741 1758 10.1016/j.biocel.2006.04.004 16740401

[B85] André S Pei Z Siebert HC Ramström O Gabius H-J Glycosyldisulfides from dynamic combinatorial libraries as O-glycoside mimetics for plant and endogenous lectins: their reactivities in solid-phase and cell assays and conformational analysis by molecular dynamics simulations Bioorg Med Chem 2006 14 6314 6326 16782346 10.1016/j.bmc.2006.05.045

[B86] Lohr M Kaltner H Lensch M André S Sinowatz F Gabius H-J Cell-type-specific expression of murine multifunctional galectin-3 and its association with follicular atresia/luteolysis in contrast to pro-apoptotic galectins-1 and -7 Histochem Cell Biol 2008 130 567 581 10.1007/s00418-008-0465-0 18597104

[B87] Shi SR Chaiwun B Young L Cote RJ Taylor CR Antigen retrieval technique utilizing citrate buffer or urea solution for immunohistochemical demonstration of androgen receptor in formalin-fixed paraffin sections J Histochem Cytochem 1993 41 1599 1604 7691930 10.1177/41.11.7691930

[B88] Szabo P Dam TK Smetana K Jr Dvoránková B Kübler D Brewer CF Gabius H-J Phosphorylated human lectin galectin-3: analysis of ligand binding by histochemical monitoring of normal/malignant squamous epithelia and by isothermal titration calorimetry Anat Histol Embryol 2009 38 68 75 10.1111/j.1439-0264.2008.00899.x 18983621

[B89] Acloque H Wilkinson DG Nieto MA In situ hybridization analysis of chick embryos in whole-mount and tissue sections Methods Cell Biol 2008 87 169 185 full_text 18485297 10.1016/S0091-679X(08)00209-4

[B90] Saunders JW Jr Bronner-Fraser M Operations on limb buds of avian embryos Methods in Avian Embryology 1996 San Diego: Academic Press 125 145 full_text 10.1016/s0091-679x(08)60626-3 8722474

[B91] Nicholson NS Panzer-Knodle SG Haas NF Taite BB Szalony JA Page JD Feigen LP Lansky DM Salyers AK Assessment of platelet function assays Am Heart J 1998 135 S170 178 10.1016/S0002-8703(98)70245-5 9588396

[B92] Pogliani E Deliliers GL Ferrari R Pozzoli E CoFrancesco E Praga C Platelet aggregometry and anti-platelet isoantibodies Haemostasis 1975 4 23 35 172412 10.1159/000214085

[B93] Hsu DK Liu FT Regulation of cellular homeostasis by galectins Glycoconj J 2004 19 507 15 10.1023/B:GLYC.0000014080.95829.52 14758074

[B94] Liao P-S Chen T-S Chung P-C A fast algorithm for multilevel thresholding J Inf Sci Eng 2001 17 713 727

