# Supplementary figures and images for: A regulatory network of two galectins mediates the earliest steps of avian limb skeletal morphogenesis
Source: BMC Dev Biol. 2011 Feb 1;11:6. doi: 10.1186/1471-213X-11-6 (PMC3042966; doi:10.1186/1471-213X-11-6)

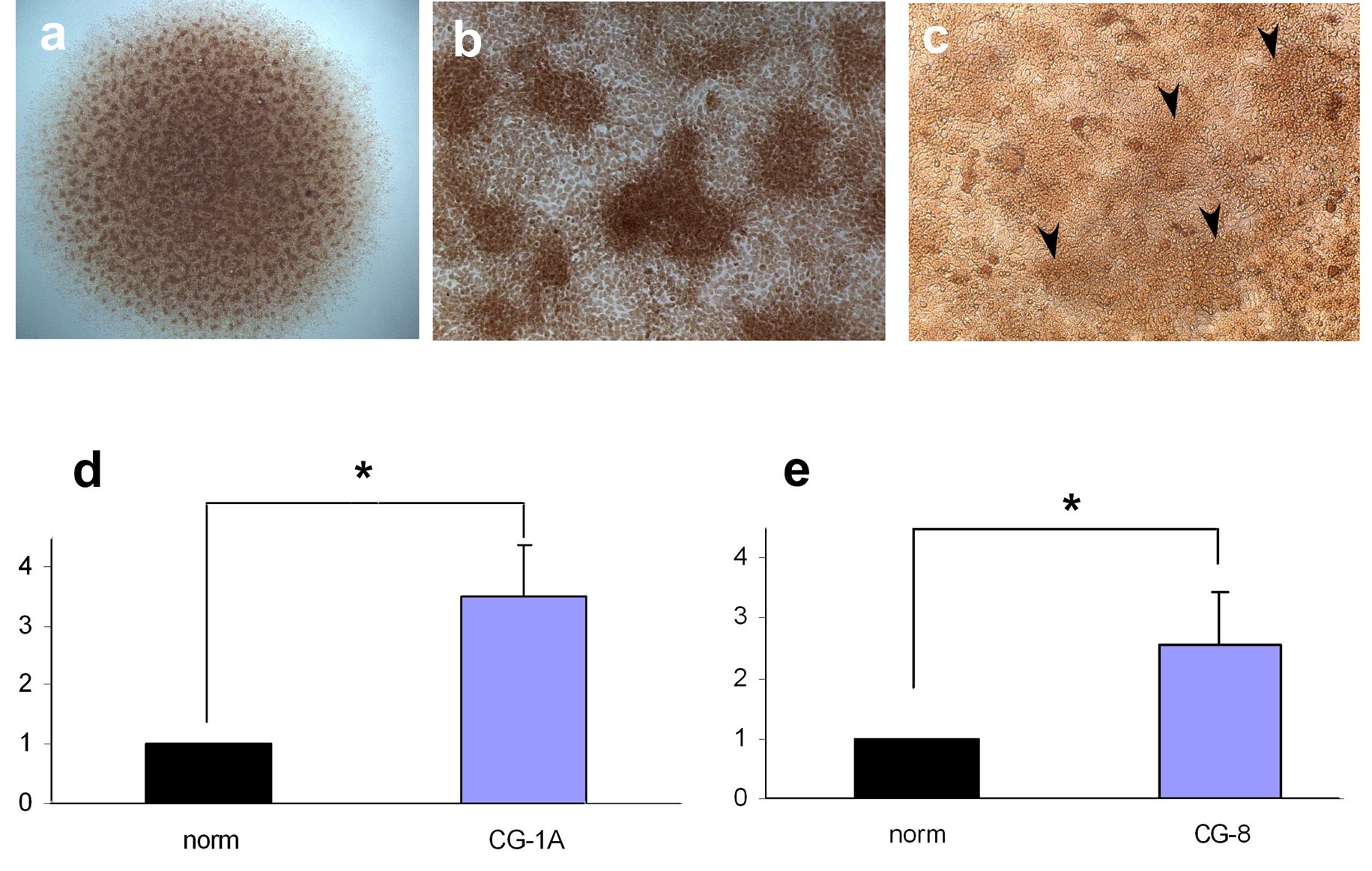

Supplement: Additional file 1 — Figure S1: CG-1A and -8 protein expression in vitro. (a-b) Spatial localization of CG-8 by indirect immunostaining in 3-day micromass leg bud mesenchymal cultures shown at low magnification (a) and high magnification (b). (c) Spatial localization of CG-8 by indirect immunostaining in 9-hour fixed cultures shows its moderately specific presence within proto-condensations (black arrowheads). (d) Graph showing condensation-specific fluorescence for CG-1A relative to DAPI fluorescence in non-condensed cells. (e) Graph showing condensation-specific fluorescence for CG-8 relative to DAPI fluorescence in non-condensed cells. Values for (d) and (e) are mean ± S.E.M. (* p < 0.05). [file 1471-213X-11-6-S1.TIFF]

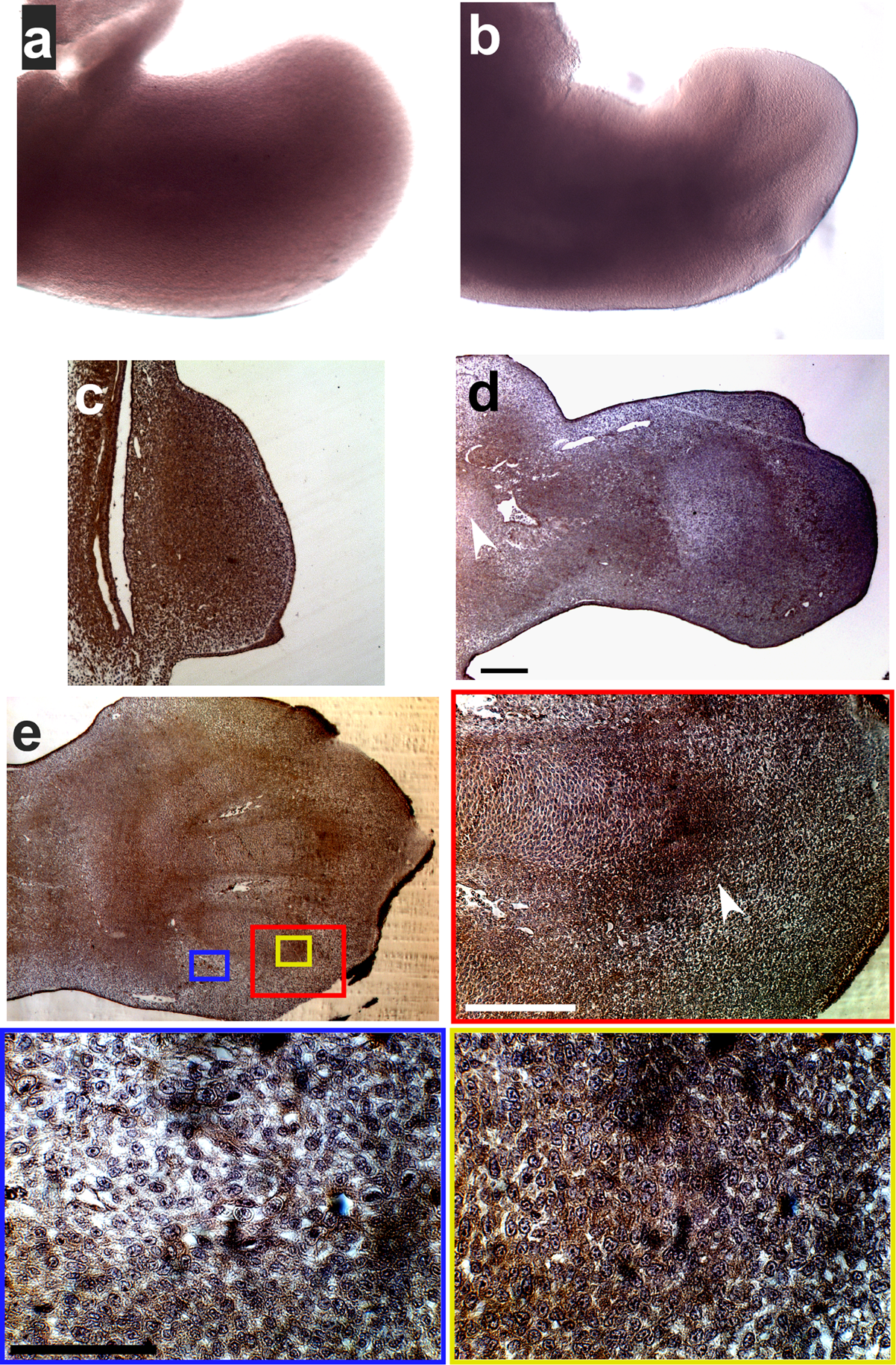

Supplement: Additional file 2 — Figure S2: Expression of CG-8 in developing chick leg buds. (a-b) Spatial expression of CG-8-specific mRNA in (a) 5-day and (b) 6-day leg buds by in situ hybridization. (c-d) Staining for CG-8 in the prospective stylopod area of a (c) 3½-day and (d) 5-day chick leg bud section, visualized by immunohistochemistry with hematoxylin counterstain. (e) Staining for CG-8 protein in 6-day leg bud section (only autopod and distal edge of zeugopodial primordia shown). The leading edge of the fourth digit primordia (outlined by the red box) shows strong crescent-shaped CG-8-specific staining (red arrowhead) at its distal edge with weak staining in cells both inside the primordia and outside. The CG-8-specific staining within the crescent fills up the extracellular space (outlined by the yellow box) whereas the extracellular space in the interdigit area is clear of CG-8 staining (outlined by blue box). The pull-out detail image in red box was photographed with 10× objective and the scale bar represents 0.2 mm. The detail images enclosed by yellow and blue boxes were photographed with 63 × oil immersion objective and the scale bar in the blue box represents 50 μm. [file 1471-213X-11-6-S2.TIFF]

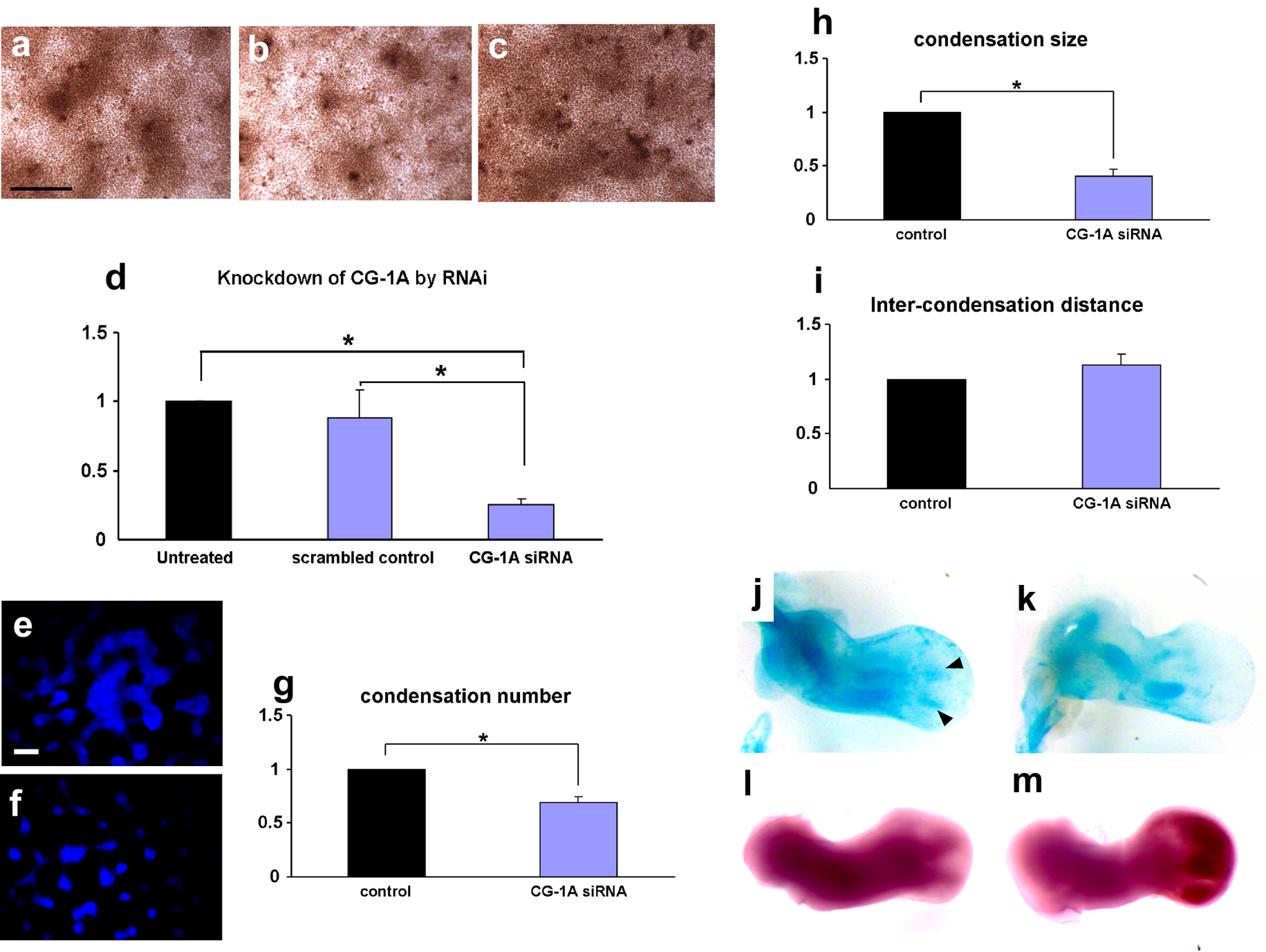

Supplement: Additional file 3 — Figure S3: Knockdown of CG-1A expression by RNAi decreases condensation number and size. (a) Indirect immunolocalization of CG-1A in a 2-day control leg culture. (b, c) Treatment of 2-day leg cultures with (b) CG-1A siRNA and (c) scrambled control oligo, both stained for CG-1A protein. (d) Relative CG-1A expression measured by qRT-PCR in untreated control, upon treatment with the scrambled control oligonucleotide, and with CG-1A siRNA. Analysis was done using Student's t-test. Results are shown as mean ± S.E.M. (*p < 0.05). (e) Control 2-day culture stained with DAPI. (f) Culture treated with CG-1A-targeting siRNA, fixed at day 2, and stained with DAPI. Photomicrographs (a-c) are at same level of magnification; bar in (a) represents 0.2 mm. Photomicrographs (d) and (e) are at same magnification and the bar in (d) represents 0.25 mm. (g) Graph showing change in mean condensation number upon knockdown of CG-1A-specific mRNA by RNAi (number in untreated control shown by black bar). (h) Graph showing change in mean condensation size upon knockdown of CG-1A-specific mRNA by RNAi. (i) Graph showing lack of significant alteration in mean inter-condensation distance upon treatment with CG-1A-targeting siRNA. The three variables (condensation number, size and inter-condensation distance) were analyzed as in the legend to Figure 5. (j, k) Untreated 7-day control wing stained with Alcian blue showing two digit primordia (j), whereas injection of CG-1A siRNA-Lipofectamine mixture into the counterpart wing on the 5th day of incubation resulted in the autopodium lacking any cartilage (k). (l, m) Untreated 6-day control wing stained for CG-1A mRNA shows a digit-like pattern in the autopodium (l) whereas injection of CG-8 in the counterpart wing on the 5th day of incubation leads to increased spatial localization and stronger CG-1A mRNA staining (m). [file 1471-213X-11-6-S3.TIFF]
